# Outcomes of Art-Based Leadership Development: A Qualitative Metasummary

**DOI:** 10.3390/bs14080714

**Published:** 2024-08-14

**Authors:** Berit Sandberg

**Affiliations:** HTW Berlin Business School, University of Applied Sciences, 10318 Berlin, Germany; berit.sandberg@htw-berlin.de

**Keywords:** art-based leadership development, art-based learning, experiential learning, competence development, leader development, leadership development, leadership skills, leadership training, qualitative metasummary, systematic review

## Abstract

Art-based leadership development, grounded in experiential learning, offers a learner-centered approach to leadership training by integrating relational, aesthetic and embodied dimensions. This systematic review investigates evidence on the outcomes of art-based leadership development, addressing the critical need for empirical validation of its effectiveness. A qualitative metasummary was employed to review 31 empirical studies published between 2008 and 2023. The studies were sourced from databases including Business Source Complete, ERIC, PsycINFO, Scopus, and Web of Science. The Quality Assessment for Diverse Studies (QuADS) tool was used to assess the studies. The analysis revealed that art-based methods significantly enhance reflective and reflexive practices, higher-order cognitive skills, emotional intelligence, and interpersonal competencies. Representing leader development, art-based initiatives facilitate holistic self-discovery and transformative shifts in mindset, offering a valuable complement to conventional skill-based approaches. Despite these promising benefits, the review highlights a need for more rigorous empirical studies, particularly longitudinal and quantitative research, to substantiate the long-term effectiveness of art-based methods.

## 1. Introduction

The expectations placed upon leadership development are contingent upon the theoretical understanding of leadership [1,2]. Theories of leadership have evolved from a focus on individual traits, skills, or behavior [3,4,5,6,7] to conceptualizations of leadership as a relational, conversational, collective, and shared phenomenon. Post-heroic theories posit leadership as a complex social process and multidirectional collective activity [8,9,10,11,12,13,14] that necessitates the process of sensemaking in dynamic, complex, and volatile environments [2,15,16,17,18,19]. In recent perspectives on leadership, the aesthetic and corporeal dimensions of leadership are acknowledged, thus embracing an embodied view of knowledge, sensory engagement, and experiential learning [20,21,22].

The differing perspectives are reflected in the fundamental distinction between leader development and leadership development as two intertwined and complementary concepts, which, however, are often not differentiated from one another [23]. While leader development places emphasis on enhancing individual qualities and related behaviors, leadership development builds on this foundation. Leadership development places the advancement and evolution of leadership abilities in both individuals and groups at the forefront, emphasizing the social processes between leaders and followers [24,25,26].

In general, traditional development programs are designed as normative trainings that are primarily expected to enhance leadership knowledge, intrapersonal competencies, and skills as well as to expand leaders’ behavioral repertoires [19,24,27,28,29,30,31]. Traditional programs tend to build on clearly defined skills and behavioral norms [16], suggesting the use of standardized tools for various leadership situations as they are following a rational, deterministic approach [32,33,34].

Against the backdrop of shifting leadership paradigms, mainstream approaches to leader or leadership development have been criticized as leader-centered, emphasizing self-development over understanding followers, and oversimplifying the complexity of leadership dynamics [32,35,36,37,38]. Traditional leadership programs frequently prioritize cognitive skills, instrumental knowledge, and logical reasoning while neglecting emotional intelligence, relational competencies, and aesthetics [39,40,41].

The overemphasis on fact-based propositional knowledge [42] comes at the cost of cultivating experiential knowledge and essential interpersonal skills [41]. The application of a technical approach to leader development, which prioritizes instrumental knowledge, superficial skills, and standardized methods, may inadvertently compromise the cultivation of humanistic values, such as compassion, empathy, and moral integrity [32,36]. Traditional leadership development programs frequently neglect the significance of experiential and relational elements within the leadership context. Such programs also fail to challenge leaders’ fundamental self-perception and moral stance, despite the fact that these elements shape leadership practice [1,36].

A counter-model of leadership development is holistic in nature. It encompasses sensemaking, embodied learning, and aesthetic reflexivity [16,33,36,41,43,44,45]. Transformative leadership development provides individualized, learner-centered opportunities for reflection and experiential learning [46,47,48] that extend beyond the competency paradigm [49]. Moreover, interventions are expected to be engaging and substantial, avoiding the pitfall of descending into mere edutainment [50,51].

The significance of leader identity in development is widely acknowledged [52,53]. Consequently, there is a pressing need for programs offering identity workspaces [54] and mindset work [1]. In the context of modern leadership, the capacity to embrace ambiguity and uncertainty is of paramount importance. This necessitates the cultivation of a growth mindset conducive to open-mindedness, adaptability, and resilience [55,56,57]. From this perspective, leadership development entails challenging mental models and questioning established behavioral patterns [1].

The integration of the arts into leadership development is expected to fulfill such expectations by challenging mainstream approaches. Although there has been a history of using arts as a metaphor for leadership (e.g., [58,59]), scholars first began linking the arts to management education [60] and leadership andragogy in the early 1990s [61,62,63,64,65]. Following the turn of the century, there was a growing interest among practitioners and researchers in art-based leadership andragogy [48,66]. It can be presumed that Romanowska and colleagues [67] were the first to introduce the term “art-based leadership development” into the scientific discourse. Nevertheless, the core concept is not necessarily labelled as art-based (e.g., [68,69]) but also referred to as art-informed [70] or art-infused [71].

Art-based pedagogy and andragogy are rooted in experiential learning [72] and are learner-centered and multimodal [73,74]. In the context of leadership development, art-based methods engage the senses through aesthetic experience, immersing learners in playful explorations of implicit leadership theories [19,75,76,77]. Art-based methods originate in artistic practice, which distinguishes them as a unique approach. Facilitators utilize artworks as a subject of reflection or engage aspiring and practicing leaders in artistic creation to explore non-artistic issues [78,79,80].

Numerous examples illustrate a form of engagement with visual and performative art that takes artworks as a starting point for inquiry and discussion [81,82,83,84]. Other interventions involve participants in creating drawings [35,69,85], collages [45], zines [86], dolls [87], masks [61,88], and statuary arts [89], usually as representations of leader identity.

Theater-based approaches, including applied drama and improvisational techniques, address interpersonal skills and an agile mindset [18,90,91,92,93,94]. The reading of literature [95,96,97,98] and poetry [99] as well as poetry writing [100] and storytelling [101] are predominantly employed to enhance creativity and provide a deeper understanding of self and human nature.

Movies and TV series offer illustrative material for reflection on leadership behavior [39,48,102,103,104]. Similarly, music [105,106], particularly coral conducting [22] and choir singing [107], as well as dance [108,109,110,111,112,113,114] provide dynamic platforms for exploring leadership and followership through collaborative practice and embodied experience.

A substantial corpus of conceptual papers and application reports has been produced which collectively asserts the benefits of art-based methods in leadership development. Art-based methods are assumed to foster cognitive leadership skills such as visioning, problem-solving, and decision-making [78]. However, their specific potential appears to lie in holistic self-discovery. Art-based leadership development is presumed to stimulate self-awareness [16], uncover subconscious behavior patterns [34,43], and make the embodied dimension of leadership accessible [115]. Art-based andragogy is posited to supplement technical leadership skills by raising aesthetic awareness, activating emotional intelligence, and fostering a transformative shift in mindset [16,20,48,69,109,116].

Nevertheless, empirical evidence supporting these notions has thus far only been subjected to systematic investigation to a limited extent [117,118]. On the one hand, there appears to be a paucity of research that extends beyond individual learner feedback and anecdotal evidence. On the other hand, there is a preference for qualitative research designs [22,75]. Those who espouse art-based andragogy maintain its efficacy, yet there has been comparatively limited research to substantiate its immediate and long-term impact [119,120,121]. In light of the growing significance of evidence-based practice in leadership development, this viewpoint is increasingly untenable [122,123,124,125]. Obscure empirical evidence and a lack of quantitative research may prevent the promising art-based approach from gaining traction [126,127].

In order to substantiate the credibility of art-based leadership development and to highlight existing research gaps, a systematic review of empirical studies on art-based leadership development was carried out. The objective of this review was to assess the empirical evidence on the outcomes of art-based leadership development from the perspective of participants. A skillset orientation to leadership development would justify the search for empirical evidence with an accountability logic calling for a measurable return on investment and quantifiable results [1,2,128,129]. In contrast, this review is intended to inform research and confirm the efficacy of art-based practice [130] without necessarily adhering to a reductionist framework that neglects the nuanced and often intangible effects of art-based methods.

In accordance with the aforementioned approach and in order to provide a comprehensive overview of the state of empirical research, this review includes quantitative, qualitative, and mixed-methods studies. Given the diversity of research approaches in art-based leadership development, the concept of qualitative metasummary by Sandelowski and colleagues [131] is employed to synthesize the findings and present a holistic understanding of the field. Details on this method will be presented in the following section.

This review did not focus on specific learning outcomes (see [132]), but considered all kinds of effects of art-based methods in leadership development as depicted in empirical studies, including participants’ immediate reactions to art-based learning environments. The findings indicate that art-based initiatives have a significant impact on enhancing reflective and reflexive practices and interpersonal competencies in learners. With a focus on leader development, art-based approaches facilitate holistic self-discovery and a transformative shift in mindset, suggesting them as an effective supplement to conventional skill-based methods. Despite the promising benefits, there is a notable deficiency in robust empirical evidence, and a need for more longitudinal and quantitative research to validate the long-term effectiveness of art-based approaches.

## 2. Materials and Methods

### 2.1. Study Design

This review adheres to the methodological framework for conducting systematic reviews as delineated by Kitchenham and Charters [133]. The approach encompasses the following stages: study selection, identification of research, quality assessment, data extraction, and data synthesis. The data extraction and synthesis are based on the principles of qualitative metasummary, a quantitatively oriented aggregation of qualitative and quantitative research findings [131]. The protocol for this systematic review was registered on INPLASY (INPLASY202460123). The reporting follows the Preferred Reporting Items for Systematic reviews and Meta-Analyses (PRISMA) statement [134].

### 2.2. Eligibility Criteria

As the concepts in question are not clearly delineated [23], this review encompasses empirical studies on leader development, leadership development, and leadership training. In order to ensure the inclusion of only those studies that are relevant to the topic at hand, research on the arts in management education and training is not considered unless leadership is explicitly mentioned. This is in accordance with the conceptual distinction between leadership and management [135,136,137].

As childhood and adulthood relate to different stages in aesthetic development, and the outcomes of child-centered and youth education are not necessarily comparable with effects on adult learners [138,139,140], the scope is on art-based andragogy, limiting the field to higher education and professional development.

The present study excluded intervention designs that are not considered art-based, such as photovoice [60,141]. Studies on leadership development in the arts and leadership pedagogy/andragogy in arts education were not considered because art-based pedagogy/andragogy, by definition, relates to transferring artistic practice to non-artistic fields [142].

The literature screening covered studies presenting effects of art-based leadership development on learners such as experience and satisfaction, competence development (knowledge, skills, attitudes), and transfer to practice [143,144].

In order to provide a comprehensive overview of robust empirical research on the subject under investigation, the review includes quantitative, qualitative, and mixed-methods studies. Papers presenting anecdotal evidence, descriptive observations, or opinion polls without scientific rigor were excluded from the sample.

### 2.3. Literature Search and Screening

A systematic search for primary research studies was conducted in electronic databases relevant to business, education, psychology, and interdisciplinary studies. The databases searched were Business Source Complete, ERIC, PsycINFO, Scopus, and Web of Science. The Boolean phrase (leadership AND (development OR training OR education OR pedagogy OR learning) AND (art OR arts OR painting OR sculpture OR music OR dance OR drama OR poetry OR movie)) was applied to titles and abstracts. The complete search strategy is displayed in Table S1. The database search was limited to articles with available abstracts.

In order to ensure a comprehensive literature review, the database search was complemented with the web-based academic search engine Google Scholar [145,146,147]. The same keywords and limiters were applied, and the results were sorted by relevance (see Table S1). Articles were selected based on titles and snippets, following a cut-off rule.

In order to ensure the quality of the research, only peer-reviewed journal articles in the English language that were published between January 2004 and December 2023 were considered. This approach takes into account the increasing body of relevant research that has been produced since the early 2000s, which was identified through a scoping search. Dissertations, book chapters, and other articles that have not undergone independent review were excluded.

**Figure 1:**
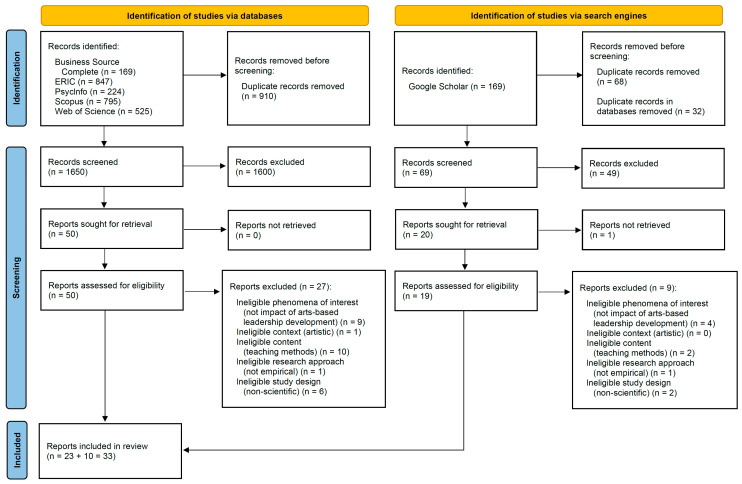
PRISMA flow diagram for literature search and selection.

The database searches and Google Scholar search together yielded an initial 1719 potentially relevant articles. Subsequently, titles and abstracts were screened against the inclusion criteria, resulting in 69 articles in total for full-text screening. After the screening process 33 articles remained for evaluation. As three reports originate from the same parent study [32,67,79], the sample includes 31 studies altogether. The search outcomes are presented in Figure 1, which follows the format of a standard PRISMA flow diagram [134].

Screening was conducted by the author and a second reviewer using a review software, the Joanna Briggs Institute System for the Unified Management, Assessment and Review of Information (JBI SUMARI) [148]. The concordance for title and abstract screening was initially established at a rate of 99%. In the event that a conflict could not be resolved through discussion, the reviewers included the relevant studies for further examination [149,150]. The full-text screening yielded a 100% match.

### 2.4. Quality Assessment

The studies included in the review were evaluated for their methodological quality, evidence quality, and reporting quality using the Quality Assessment for Diverse Studies (QuADS) tool [151,152,153]. The Quality Assessment for Diverse Studies (QuADS) tool was developed for use in systematic reviews that encompass a broad range of study designs. Its integration enables a consistent and comprehensive evaluation, facilitating comparison [151].

QuADS is a thoroughly tested, valid appraisal instrument that has proven its informational and reliability value in the field of psychology [151]. As art-based leadership development draws on psychological constructs to facilitate holistic growth in leaders, QuADS is an appropriate tool. It has been employed in multiple reviews of educational interventions (e.g., [154,155]) and is a useful tool for measuring the effectiveness of such initiatives.

The QuADS framework comprises 13 criteria that encompass a comprehensive range of elements pertaining to the quality of research design and transparency of reporting. These include research objectives, theoretical framework, sampling methodology, data collection procedures, data analysis techniques, and limitations. Each criterion is evaluated on a scale of 0 to 3, with a maximum total score of 39 for each study [153].

The author and a second reviewer independently assessed all studies for quality based on the QuADS assessment matrix [153]. We followed the recommended iterative process and began with an arbitrary selection of two methodologically distinct studies. To establish a shared understanding of the criteria, we discussed our scorings and repeated the comparison based on another three studies before we assessed all remaining studies [152]. Initially, the interrater-agreement percentage was 97.9% on average for the entire sample. Any discrepancies were discussed and resolved through mutual agreement [149]. The assessment results are presented in Table 1.

As the QuADS criteria are not weighted and there is no defined threshold score to classify a study as high or low quality, the assessment results will be discussed with a focus on criteria that are particularly relevant for demonstrating the effectiveness of art-based methods [151,152]. These are the QuADS criteria no. 4, goal-adequate study design, no. 5, appropriate sampling, no. 7, data collection method, and no. 11, method of data analysis. These aspects have a particular impact on a study’s rigor and the generalizability of its findings [156].

In order to ensure a comprehensive analysis and achieve a more nuanced understanding of the evidence base, all studies were retained for further analysis, regardless of their quality [131,157,158].

### 2.5. Data Extraction

In the field of data analysis, large language models, such as OpenAI’s GPT series, have demonstrated comparable performance to humans [159]. These models significantly enhance the literature review process through the accurate and efficient summarization and categorization of studies [160,161]. Therefore, data were extracted using GPTs to create a data-extraction matrix. This matrix includes the following variables: author(s), year of publication, geographic region of first author, intervention type, study design, participant characteristics and sample size, data collection, data analysis, aim of intervention, and key findings on outcomes. The data-extraction matrix is presented in Table 2.

The relevant data on the study characteristics and research design were extracted with the GPTs LitReviewGPT [162] and AskYourPDF Researcher [163] in parallel. The results were compiled and verified for accuracy in full by the author [157], demonstrating high correspondence with the wording of the studies. In certain instances, there was a discrepancy between the reported study type and the actual implementation. For instance, some studies that were labeled as grounded theory by the authors actually aligned more with qualitative descriptive studies due to the absence of essential grounded theory processes, such as theoretical sampling [164]. In such cases, the classification of the study design and data analysis was corrected by the author and then verified by a third reviewer.

The data on outcomes were extracted with the GPT AskYourPDF Researcher [163] and were checked for accuracy by the author and the second reviewer after a thorough reading of all studies [165]. Gradual prompt engineering was employed to ensure that the generated excerpts were based on empirical findings unique to the study in question, rather than the authors’ discussion of prior research [131]. For each study and outcome, the GPT generated a snippet and an even more condensed catchphrase. Any catchphrases that were incomprehensible outside the context were slightly adjusted. Any missing results were added with snippets and catchphrases quoted verbatim from the studies.

The 33 outcome summaries constituted the foundation for a codebook utilized in the course of data analysis. All codes (catchphrases) and code descriptions (snippets) are presented in Supplement 3 (Supplementary Materials). The codes are included in the data-extraction matrix (see Table 2).

**Table 1:** QuADS quality assessment of studies. Source [153].

Author(s)	1	2	3	4	5	6	7	8	9	10	11	12	13	Total Score/39
Andenoro & Ward (2008) [166]	2	3	3	1	1	1	1	1	1	1	1	1	0	17
Cranston & Kusanovich (2013) [167]	1	2	1	2	0	1	1	0	1	1	2	0	0	12
Cranston & Kusanovich (2014) [168]	2	2	1	1	0	0	1	1	1	1	2	0	0	12
Dennis (2014) [169]	1	2	1	1	0	0	1	0	0	0	0	0	0	6
Feltham (2012) [170]	1	2	2	1	0	1	0	0	1	0	2	0	0	10
Firing et al. (2022) [171]	1	2	3	3	1	1	3	3	1	2	3	0	1	24
Garavan et al. (2015) [69]	3	1	3	3	3	0	3	1	3	0	3	0	3	26
Harz et al. (2023) [172]	2	1	3	3	1	3	3	3	3	3	3	0	3	31
Hirsch et al. (2023) [57]	2	2	2	3	0	0	2	2	0	0	1	0	0	14
Hurdle & Greenhaw (2023) [173]	3	3	3	3	1	0	3	2	3	1	3	0	3	28
Kaimal et al. (2014) [174]	1	1	3	3	1	1	3	2	1	0	3	0	1	20
Kaimal et al. (2016) [175]	1	1	3	3	1	0	3	2	1	0	3	0	1	19
Katz-Buonincontro (2011) [50]	1	1	1	0	1	2	0	1	0	1	2	0	0	10
Katz-Buonincontro & Phillips (2011) [176]	1	2	3	3	0	1	3	0	1	2	3	0	1	20
Katz-Buonincontro et al. (2015) [177]	1	2	3	0	1	0	0	0	1	1	1	0	1	11
Kilic (2023) [40]	3	3	3	3	2	3	3	3	3	1	3	0	0	30
Leonard et al. (2013) [106]	2	1	3	2	0	0	2	2	2	3	3	2	0	22
Medeiros et al. (2012) [178]	0	2	3	2	0	0	0	0	0	0	0	0	0	7
Munro et al. (2015) [179]	2	2	3	2	1	3	3	1	0	0	0	0	1	18
Parush & Koivunen (2014) [68]	2	2	3	3	0	0	3	1	0	0	3	0	0	17
Peña & Grant (2019) [180]	3	3	2	2	1	0	2	1	0	0	3	0	3	20
Rajendran & Andrew (2014) [51]	1	2	3	2	0	0	2	1	1	0	3	0	1	16
Romanowska et al. (2011) [67]	0	3	3	3	3	2	3	3	3	1	3	1	3	31
Romanowska et al. (2013) [32]	3	3	3	3	3	2	3	3	3	1	3	1	3	34
Romanowska et al. (2014) [79]	3	2	3	3	3	2	3	3	3	2	3	1	3	34
Sandberg et al. (2023) [181]	3	2	3	2	2	2	3	3	3	2	3	0	3	31
Schyns et al. (2013) [35]	3	2	1	3	0	3	3	2	1	2	3	0	0	23
Singh & Widén (2020) [182]	0	2	3	2	0	0	2	2	1	0	1	0	0	13
Sutherland (2012) [183]	3	1	2	3	0	0	3	3	1	2	3	0	1	22
Sutherland & Jelinek (2015) [22]	3	3	3	3	2	0	3	3	1	3	3	0	1	28
Winther (2018) [184]	1	3	2	1	0	2	2	1	1	0	0	0	0	13
Winther & Højlund-Larsen (2022) [185]	2	2	2	2	0	3	2	1	1	1	1	0	0	17
Woods et al. (2023) [45]	3	2	3	3	0	3	3	3	1	3	3	0	1	28
Note. QuADS criteria: 1. Theoretical or conceptual underpinning to the research. 2. Statement of research aim/s. 3. Clear description of research setting and target population. 4. The study design is appropriate to address the stated research aim/s. 5. Appropriate sampling to address the research aim/s. 6. Rationale for choice of data collection tool/s. 7. The format and content of data collection tool is appropriate to address the stated research aim/s. 8. Description of data collection procedure. 9. Recruitment data provided. 10. Justification for analytic method selected. 11. The method of analysis was appropriate to answer the research aim/s. 12. Evidence that the research stakeholders have been considered in research design or conduct. 13. Strengths and limitations critically discussed.														

**Table 2:** Data-extraction matrix.

Author(s)	Approach	Study Design	Sample	Data Collection	Data Analysis	Aims of Intervention	Outcome
Andenoro & Ward (2008) [166] USA	Watching movies	Case study	Undergraduate students *n* = 31	Focus groups	Content analysis Constant comparative method	Critical thinking skills Leadership competencies	Enhanced engagement Improved critical thinking skills Broader leadership perspective Real-world application of theories Increased reflective thinking Enhanced empathy High satisfaction with course design
Cranston & Kusanovich (2013) [167] Canada	Applied drama	Qualitative descriptive study	School leaders, leaders of educational institutions, teachers, nascent educational administrators *n* = 14	Pre- and post-workshop open-ended questionnaires Nonparticipant observer field notes	Content analysis Recursive analysis	Ethical decision making Understanding of school leadership	Enhanced empathy Increased reflective thinking High satisfaction with course design Improved ethical understanding Real-world application
Cranston & Kusanovich (2014) [168] USA	Applied drama	Qualitative descriptive study	School leaders, teachers in leading roles *n* = 16	Participant journals	Content analysis Recursive analysis	Ethical decision making Understanding of school leadership	Enhanced ethical understanding Increased empathy Improved reflective thinking Greater engagement Relevance of risk-taking Development of practical skills Enhanced collaborative decision-making High satisfaction with course design
Dennis (2014) [169] Australia	Dance Movement-based practice Improvisation	Qualitative action research study	Emerging leaders Four programs with *n* = 24–40 each	Observation Interviews Participant reflective comments	Phenomenological analysis	Relational and task-based leadership capabilities Capacity to tolerate ambiguity and uncertainty Motivation and productivity	Enhanced self-awareness Improved interpersonal efficacy Emotional transformation Increased personal agency Reflective practice Cultural exploration
Feltham (2012) [170] UK	Applied drama	Qualitative descriptive study	Individuals connected with the training event *n* = 5	In-depth interviews	Thematic analysis	Leadership skills	Improved interpersonal skills Enhanced reflective practice Increased empathy Enhanced emotional intelligence Behavioral change Increased confidence Stress management Improved well-being
Firing et al. (2022) [171] Norway	Applied drama	Case study	RNoAFA cadets *n* = 14 (thereof informants *n* = 8)	In-depth interviews Participatory field observation	Thematic analysis Constant comparative method	Coping with complexity and volatility	Transformative learning Holistic identity development Increased empathy Enhanced emotional awareness Enhanced social awareness Community building Overcoming anxiety Managing uncertainty
Garavan et al. (2015) [69] UK	Drawing	Quasi-experimental study	MNC leaders *n* = 164	Pre-test post-test surveys	Statistical analysis	Emotional intelligence Leader identity Openness to experience Feedback orientation	Improved emotional intelligence Enhanced leader identity Increased feedback orientation
Harz et al. (2023) [172] USA	Listening to music Artist talk	Qualitative descriptive study	Medical and dental students *n* = 122	Post-test survey	Content analysis Statistical analysis	Awareness of empathy, human dignity, communication, and teamwork	High satisfaction with course design Enhanced empathy Holistic perspective Improved reflective practice Appreciation for the arts Awareness Broader perspective Wellbeing
Hirsch et al. (2023) [57] USA	Watching movie clips Clay molding	Qualitative descriptive study	Organizational leaders from business and law enforcement *n* = 10	Participant reflective journals Interviews	Thematic analysis	Negative capability	Development of negative capability Increased self-awareness Emotional regulation Integration of body and mind Enhanced reflectivity Utilization of creative processes
Hurdle & Greenhaw (2023) [173] USA	Watching movies	Case Study	Students *n* = 9	Participant-written assignments	Content analysis Concept coding	Understanding the stages of group development	High satisfaction with assignment Effective learning tool Vicarious learning
Kaimal et al. (2014) [174] USA	Music-making Tango dance	Case study	School leaders, principal interns *n* = 20	Observation Participant reflective papers Interviews	Thematic analysis	Leadership-arts integration Creative thinking enhancement	Enhanced reflective practice Increased creativity Increased innovation Empowerment Increased agency Broadened perspectives on leadership
Kaimal et al. (2016) [175] USA	Engaging with paintings Drawing	Case study	Principal interns *n* = 14	Feedback survey Observation Interviews	Thematic analysis	Creativity Imagination	Enhanced reflective practice Increased empathy Broadened perspectives on leadership Empowerment Transfer to professional context
Katz-Buonincontro (2011) [50] USA	Improvisational theatre	Case study	Educational leadership students *n* = 30/11	Interviews Observations Improv videos Extant documents	Grounded theory Constant comparative method	Leadership empowerment	Emotional catharsis Enhanced empathy Heightened sensory perception Reflective thinking Increased creativity Sense of community
Katz-Buonincontro & Phillips (2011) [176] USA	Drawing Pottery making Improvisation Improvi- sational theatre	Comparative case study	Educational leadership doctoral students *n* = 21 Educational leadership students *n* = 140	In-depth interviews Participatory field observations Participant reflective journals Workshop photographs Blackboard discussion threads Course evaluations Videos of improv role-plays	Grounded theory Constant comparative method	Problem-solving skills	Enhanced reflectivity Improved problem-solving skills Increased creativity Increased risk-taking Heightened sense of visual perception
Katz-Buonincontro et al. (2015) [177] USA	Drawing Collage Photo- captioning Cabinetry Pottery Viewing visual art Improvi-sational theatre	Comparative case study	Educational leadership students *n* = 77	Participant reflective journals Class discussions Participant artworks Transcriptions of improv exercises	Arts-based inquiry Thematic analysis	Leader identity Vision-building skills Problem-solving skills	In-depth personal reflection Increased observational skills Encouraged risk-taking Reconnection with creativity Leadership paradigm definition Enhanced understanding Insight into leadership practice
Kilic (2023) [40] Turkey	Drawing Role-play Story- telling Music Dance	Mixed-methods action research study Quasi-experimental sub-study	Business leaders *n* = 15	Pre-test post-test inventory Pre-test post-test questionnaire Pre-test post-test Affect Grid Interviews Observation Participant drawings	Statistical analysis Thematic analysis	Creativity Communication skills Resilience Social sensitivity	Enhanced creativity Improved communication skills Healing effect Increased social sensitivity Enhanced reflectivity Sense of community
Leonard et al. (2013) [106] UK	Gamelan music-making	Qualitative descriptive study	Post-Qualification students (nurses, social workers), trainers *n* = 31	Post-intervention questionnaire	Thematic analysis	Teamwork Collaboration Creativity Distributive and participatory leadership skills	Enhanced reflective learning Increased emotional engagement Improved teamwork Improved collaboration Increased willingness to take risks Learning transfer to real world
Medeiros et al. (2012) [178] Brazil	Reworking paintings	Quantitative descriptive study	Medical students *n* not reported	Post-test questionnaire	Statistical analysis	Humanist skills	Enhanced ethical humanist skills Improved teamwork Improved leadership Improved communication
Munro et al. (2015) [179] UK	Applied drama	Quantitative exploratory study	Managers *n* not reported	Pre-test post-test inventory Pre-test post-test questionnaire	Statistical analysis	Communication skills	Heightened emotional awareness Increased emotional competency Increased awareness of cognitive and sensory preferences Enhanced communication effectiveness Adaptability in communication modes
Parush & Koivunen (2014) [68] Finland	Choral conducting	Case study	Managers, conducting students *n* not reported	Observations Interviews Feedback questionnaires	Thematic analysis	Self-exploration Self-improvement	Heightened aesthetic pleasure Memorability Increased self-confidence Increased risk-taking
Peña & Grant (2019) [180] USA	Painting	Qualitative phenomenological study	MBA students *n* not reported	Participant reflective journals	Narrative analysis	Self-exploration	Disorienting dilemma Sense-making Self-awareness Self-efficacy
Rajendran & Andrew (2014) [51] Australia	Watching movies	Qualitative action research study	Management students *n* = 30	Focus groups	Thematic analysis Constant comparative method	Knowledge on leadership theory Cultural understanding Open-mindedness Reflection	Enhanced memorization Improved understanding Contextual understanding Learner autonomy Engagement and motivation Practical learning experience
Romanowska et al. (2011) [67] Sweden	Witnessing performance art	Experimental study	Managers and their subordinates *n* = 48 + 192 = 240 at baseline	Pre-test post-test questionnaires Pre-test post-test blood samples	Statistical analysis	Mental and biological stress	Improved mental health Enhanced coping strategies Better performance-based self-esteem Favorable biological outcomes Reduction in stress indicators
Romanowska et al. (2013) [32] Sweden	Witnessing performance art	Experimental study	Managers and their subordinates *n* = 48 + 192 = 240 at baseline	Pre-test post-test inventory Pre-test post-test questionnaires	Statistical analysis	Sense of coherence Agreeableness Capacity to cope with stress Laissez-faire leadership	Improved mental resilience Enhanced pro-social behavior Reduced passive leadership Better stress management
Romanowska et al. (2014) [79] Sweden	Witnessing performance art	Experimental study	Managers and their subordinates *n* = 48 + 192 = 240 at baseline	Pre-test post-test questionnaires Pre-test post-test blood samples	Statistical analysis	Self-awareness Humility Capacity to cope with stress Laissez-faire leadership	Improved self-awareness Enhanced perceptual alignment Reduction in passive leadership Better stress management Positive impact on subordinates Improved leader performance
Sandberg et al. (2023) [181] Germany	Dance	Mixed-methods study Quasi-experimental sub-study	Managers *n* = 23/14	Pretest-posttest questionnaires Interviews	Statistical analysis Thematic analysis	Attention Presence Mutual engagement Resilience	Improved physical presence Enhanced nonverbal communication Positive aesthetic experience Increased sensitivity Successful learning transfer
Schyns et al. (2013) [35] UK	Drawing	Qualitative descriptive study	Undergraduate postgraduate and executive students *n* = 138 drawings created by participants	Drawings	Content analysis	Reflection on leadership Self-awareness and social awareness about implicit leadership theories	Heightened self-reflection Enhanced self-awareness
Singh & Widén (2020) [182] USA	Watching movies	Qualitative descriptive study	Library and information science students *n* = 101	Participant reflective papers	Content analysis	Learning about leadership concepts and theories Critical thinking skills	Changed leadership perspectives Improved critical thinking Engaged learning Recognition of essential leadership traits Practical application
Sutherland (2012) [183] Slovenia	Choral conducting	Qualitative grounded theory study	Executive MBA students *n* = 24	Participant reflective essays	Grounded theory Constant comparative method	Not reported	Enhanced reflexivity High aesthetic engagement Increased emotional awareness Improved self-awareness Memorable learning experiences Reconsidering future leadership practice
Sutherland & Jelinek (2015) [22] Slovenia	Choral conducting	Case study	Executive MBA students, early career managers *n* = 15	Observations and conversations with facilitators Participant observation Interviews with participants	Grounded theory	Not reported	Heightened awareness of relational dynamics Deeper understanding of power and responsibility Long-term impact on professional practice Emotional engagement Reflective practice Aesthetic experience Sense-making Enhanced humanistic qualities
Winther (2018) [184] Denmark	Dance	Qualitative performative study	Pre-service teachers *n* = 21	Written student experience reports Documentary film	Phenomenological thematic analysis	Somatic awareness Creativity Leadership	Increased self-confidence Increased sensitivity Improved self-contact Somatic awareness Embodied leadership
Winther & Højlund Larsen (2022) [185] Denmark	Dance	Qualitative phenomenological study	Leaders from diverse fields *n* = 9	Written participant reflections	Phenomenological analysis	Embodied leadership competence	Increased embodied leadership competence Enhanced emotional awareness Improved reflexivity Enhanced communication skills Development of relational skills Sustained growth Improved self-contact Somatic awareness
Woods et al. (2023) [45] UK	Collage-creation Gesture- response	Qualitative action research study	Educators *n* = 44	Interviews Participant written reflections Photos of collages Workshop videos Field notes	Thematic analysis	Capacity for distributed leadership Aesthetic qualities	Enhanced aesthetic awareness Improved collaborative leadership capabilities Transformative learning Increased reflexivity Self-awareness Interpersonal awareness Widening perspectives on leadership Increasing capacity for pro-active agency

### 2.6. Data Synthesis

The process of mixed research synthesis is inherently challenging due to the complex task of comparing and combining the diverse methodologies and topical differences inherent in qualitative and quantitative studies [131]. Qualitative metasummary represents a unique approach to integrating qualitative and quantitative research findings on a topic by extracting descriptive findings from diverse studies and aggregating them through a quantitatively oriented approach [131]. The method provides a degree of rigor that allows for the generalization of results [186].

The synthesis of outcome findings was based on their codes. Identical codes and codes with the same meaning were grouped to sub-themes, adjusted for redundancies, and referenced with the study from which they were derived [131]. Based on the similarities in content, the resulting 155 sub-themes were aggregated to 27 themes and finally organized into 11 overarching main themes [187]. To prevent bias and ensure plausibility, the author and Reviewer 3 discussed the abstraction process [188,189]. The resulting thematic framework is presented in full in Table S2. Table 3 provides an example of the hierarchy for the main theme “higher-order cognitive skills”.

To assess the relative magnitude of themes, their frequency effect sizes were calculated by taking the number of reports representing a theme and dividing it by the total number of reports in the sample (33 reports). This resulted in the percentage of articles reporting a certain outcome. Articles derived from a common parent study representing a duplication of the same theme, namely Romanowska and colleagues [32,67,79], were counted only once in the numerator and denominator [131]. The frequency effect sizes are reported in Table 4.

To ascertain which studies contributed to the identified themes, the intensity effect size of each study was determined in two ways. The first was the frequency effect size A, calculated by dividing the number of themes with frequency effect sizes ≥25% contained in a study by the number of themes with frequency effect sizes ≥25% across all studies (5 themes). This value indicates the relative contribution of a study to the most significant findings across all studies. The frequency effect size B was derived by dividing the number of themes contained in a study by the total number of themes across all studies (27 themes). This value indicates how many themes are captured within the study [131,190]. The intensity effect sizes are listed in Table 5.

## 3. Results

### 3.1. Study Characteristics

#### 3.1.1. Research Setting and Sample Characteristics

The 33 reports and 31 studies, respectively, in the sample span from 2008 to 2023. There has been a slight increase in study intensity in recent years, with seven studies published in 2022 and 2023. The studies originate from North America (13 studies, thereof 12 from the US), Europe (17, thereof 6 from the UK), Australia (2), and South America (1).

The reports encompass a diverse array of art-based approaches to leadership development. The majority of interventions employed a combination of various art forms [40,45,57,174,176,177].

Five articles report on workshops where participants created paintings [178,180], made drawings [35,69,175], or engaged with paintings as objects for reflection [175]. Another five studies focus on applied drama [167,168,170,171,179]. One study addresses improvisational theater [50].

Three reports are based on a parent study where participants witnessed performance art [32,67,79], the only study in the sample referencing literature and poetry. Four studies examine the effects of dance and movement-based practices on leaders [169,181,184,185].

One study examines the impact of joint music-making [106], three discuss leaders’ experiences with choral conducting [22,68,183], and another focuses on a listening experience and an artist talk with a musician [172]. Finally, four articles investigate the learning outcomes of watching movies [51,166,173,182].

In fewer than half of the studies (12/31), professionals in a lead role were involved. The majority of interventions were designed as single sessions, with a maximum duration of two-days. A total of six studies included participants in intermittent sessions over time spans of two to 18 months [32,67,79,166,171,176,179,185] (see Table 6). Empirical research on the use of movies as teaching material is exclusively derived from student participant groups.

#### 3.1.2. Study Design and Quality

Empirical research on art-based leadership development is predominantly qualitative (see Table 6). The 25 qualitative studies examined cover various approaches, including, among others, ten case studies, two phenomenological studies [180,185], and one performative study [184]. The review encompasses four quantitative studies: one experimental study that generated three reports [32,67,79], one quasi-experimental study [69], and two quantitative descriptive studies [178,179]. Furthermore, two mixed-methods studies with quasi-experimental sub-studies [40,181] are included in the sample.

The typical methodology employed by researchers is to integrate their inquiries into existing training programs and to rely on convenience sampling (10 studies) or purposive sampling (12 studies). It is a relatively uncommon practice among researchers to source participants independently (e.g., [40,67,181]). Two quantitative studies employed random sampling and a comparative group [32,67,69,79], while the others followed a single group design. The reported sample sizes for qualitative studies ranged from 5 to 140, while those for quantitative (sub-)studies ranged from 15 to 240. 

With the exception of Romanowska and colleagues [32,67,79], which included biometric measures, all studies were based on self-reported and/or observational data. The experimental study conducted by Romanowska and colleagues [32,67,79] is the only one that considered both the leader and follower perspective.

The majority of researchers collected qualitative data during the sessions (nine studies) or after the intervention (nine studies). With the exception of one study [178], quantitative and mixed-method studies presented baseline and post-test data. The sample includes six longitudinal studies, three of which are qualitative [22,57,106] and three of which are quantitative (sub-) studies [32,67,69,79,181] with a follow-up period of between six weeks and 18 months post-intervention.

The quality assessment of the included studies is presented in Table 1. The QuADS quality scores exhibited considerable variability, ranging from 6 to 34 of a possible 39 points, with a mean and median score of 20 across all studies (see Table 7). Qualitative studies range from 6 [169] to 31 [172] with a mean of 18. Quantitative studies achieve higher values. Their quality ranges from 7 [178] to 34 [32,79] with a mean of 25 (29 without the outlier). Mixed-method studies score 30 [40] and 31 [181].

The studies examined exhibited appropriate study designs, data collection methods, and data analysis techniques, regardless of the research approach. Lower quality assessment is generally related to the aforementioned suboptimal sampling and a lack of reporting around recruitment data and the data collection procedure, as well as the rationale for data collection and data analysis. Three research projects considered stakeholders in the design or conduct of the study.

In general, empirical research on art-based leadership development is well-founded in theory or viable concepts. A number of studies make reference to philosophy or learning theory, citing concepts such as aesthetic experience [191] and embodiment [192], experiential learning [72], and transformational learning [193]. Seven studies out of 31 align their research questions with leadership concepts, including leadership theories and concepts in general [182], laissez faire leadership [32,79], distributed leadership [45,106], artful leadership [181], and embodied leadership [184,185].

### 3.2. Outcomes

#### 3.2.1. General Findings

The findings presented in the reports were grouped into 11 main themes and 27 related themes (see Table 4). The main themes identified are reflective and reflexive practices, higher-order cognitive skills, and sense-making (see Section 3.2.2); emotional development and personal growth, sensory and experiential awareness (see Section 3.2.3); interpersonal and social competencies (see Section 3.2.4); adaptive resilience, and comprehensive leadership development (see Section 3.2.5); learner engagement and satisfaction, learning process, and transfer success (see Section 3.2.6).

As demonstrated by the effect sizes presented in Table 5, qualitative studies contribute more significantly to impactful findings and offer broader thematic coverage than quantitative studies. Although they are of higher quality (see Table 1 and Table 5), quantitative studies tend to have lower intensity effect sizes in both instances: contributing to the most impactful findings (intensity effect size A) and in terms of overall thematic contribution (intensity effect size B). While lower-quality studies may contribute to some high-impact findings, studies with higher methodological rigor generally dominate in both impact and thematic breadth. A considerable proportion of frequent findings are supported by a combination of high- and low-quality studies.

With the exception of four reports, the analysis of findings suggests that the art-based sessions were a complete success. Woods and colleagues [45] observe that the impact of the art-based approach on participants varied. For some, the art-based approach had no discernible effect [182]. Studies employing statistical analysis and appropriate reporting indicate that, contrary to the intended outcome, the sessions did not result in a notable change in openness to experience [69], in the way leaders interacted with others, nor in resilience [181].

#### 3.2.2. Cognitive and Reflective Skills

The most frequently cited effect of arts-based methods in leadership development is an increase in reflective and reflexive practices. In fact, almost every second study (15/31) reports an enhanced capacity for reflection and reflexivity among participants. While reflection involves processes of introspection and self-examination, reflexivity encompasses an awareness of the broader relational contexts that influence oneself and an appreciation for the social realities of others [194]. The art-based approach facilitated participants’ capacity for critical reflection on their actions and decisions. Participants developed enhanced reflexive capabilities, enabling them to engage in a more rigorous evaluation of their leadership practices.

A total of ten studies have documented an increase in higher-order cognitive skills, including reflective thinking, critical thinking skills, and creativity. Eight studies have referred to various aspects of sense-making, including a deeper understanding, a change of perspective, and holistic identity development. The interventions enabled participants to analyze and evaluate specific experiences and information and to make sense of complex and ambiguous situations, thus contributing to effective problem-solving and decision-making [195].

#### 3.2.3. Experiential and Emotional Development

Twenty studies were allocated to the main theme of “emotional development and personal growth”. Art-based methods have been found to have an impact on self-awareness, emotional awareness, and emotional transformation, which points to an increase in emotional intelligence [196]. As a consequence of the interventions, participants demonstrated enhanced capacity to regulate their emotional responses and an augmented ability to express emotions. Many gained a sense of personal agency, feeling more confident in their abilities to influence and drive change.

Seven studies reported that participants exhibited heightened awareness and sensitivity in terms of aesthetic, somatic, or visual perceptiveness. This heightened sensitivity and perceptiveness to sensory stimuli, coupled with an openness to new experiences, constitutes sensory and experiential awareness [197].

#### 3.2.4. Interpersonal and Social Competencies

Another significant main theme is “interpersonal and social competencies”, with 19 studies in total. Interpersonal skills and communication skills are conceptualized as interpersonal competencies that enable individuals to interact with others one-on-one or in small groups. Social competencies, including empathy, social sensitivity, and prosocial skills, refer to a broader set of skills and abilities that are needed to navigate within a larger social context [198]. The empirical findings indicate that methods based on performance arts, in particular, foster interpersonal skills and increase proficiency in both verbal and nonverbal communication. Participants expressed appreciation for the collaborative learning environments that some interventions created. They observed enhanced collaborative efforts and team cohesion. Several studies demonstrate that participants developed a greater ability to empathize with others.

#### 3.2.5. Adaptive and Resilient Leadership

Individual adaptive resilience highlights the capacity to adapt to changing circumstances while maintaining a sense of agency and control [199,200]. A limited number of studies have examined this aspect of leader development. Five studies have reported that art-based practices have encouraged participants to take more risks in their leadership practices. Two studies have supported the idea that this approach helps leaders develop negative capability, which is the ability to embrace uncertainty, doubt, and ambiguity without the need for clear answers or logical resolution [201]. Four studies have demonstrated a positive impact of arts-based methods on mental health, including improved stress management and well-being.

The implementation of art-based methods has been found to have a notable impact on the mindset of participants, resulting in a broader and more nuanced perspective on leadership, enhanced leader identity, and improved performance. Additionally, there was a significant improvement in embodied leadership. These findings are derived from a total of 12 distinct studies.

#### 3.2.6. Learning Experience and Transfer

The art-based methods were notably distinct from approaches to leadership development that learners had previously encountered [166,167]. While some participants found the different nature of the assignments enjoyable [175], others were significantly challenged by them, perceiving them as an “emotional bomb-shell” [171] (p. 336). For many, the art-based approaches engendered a “feeling of being far outside one’s comfort zone” [171] (p. 337) (see also [45,68,183,184,185]). The findings indicate that there is a disorienting dilemma present in the learning process [180,181].

The art-based interventions yielded high levels of learner engagement and satisfaction, with positive feedback on the aesthetic experience. Learners found the art-based sessions more engaging than traditional lecture-based courses. Eleven studies demonstrate the effectiveness of art-based interventions in engaging learners and enhancing satisfaction.

In seven studies, the learning process is described as transformative or comprehensive, indicating that there have been sustainable shifts in self-perception, understanding of leadership roles, and professional practice that are based on memorable experiences.

Leaders perceived the art-based sessions as distant from their everyday professional lives [68], which presented a challenge for some in transferring insights to their individual leadership practices [22]. However, one-third of the reports indicate that learners successfully transferred their insights and acquired skills to their professional contexts. Three studies demonstrate sustainable transfer success in follow-up assessments conducted four to 18 months post-intervention [22,32,67,79,181]. One longitudinal study provides substantial evidence that art-based leadership development leads to behavioral change [32,79].

## 4. Discussion

### 4.1. Effectiveness of Art-Based Leadership Development

The objective of this systematic literature review and metasummary was to identify, critically evaluate, and synthesize the evidence pertaining to the effectiveness of art-based leadership development. The 31 studies reviewed encompass a diverse range of art forms, including visual arts, applied drama, dance, music, and movies. The impact of art-based leadership development can be discerned across a variety of leadership aspects, regardless of the art form. The evidence indicates a multitude of effects.

Art-based methods have been found to significantly enhance reflective and reflexive practices among participants. The ability to critically reflect on actions and decisions, and to develop deeper reflexive capabilities, is a common outcome. The impact of art-based methods on emotional development and personal growth seems profound. Participants demonstrated increased emotional intelligence, self-awareness, and the ability to regulate emotional responses. Art-based methods also fostered significant improvements in interpersonal and social competencies. Enhanced communication skills, empathy, and collaboration are frequently reported outcomes. The advancement of these abilities through artistic processes indicates a holistic transformation in the way participants think, feel, and interact with their surroundings.

Empirical research on leadership development typically ignores collective outcomes, focusing instead on effects at the individual and team levels [202]. This trend is also evident in studies on art-based leadership development. The art-based leadership development that is currently evidenced does not imply transcending leaders’ development in leader–follower dynamics and organizational development. The objectives and outcomes of interventions are limited to an individual perspective, with a focus on personal development and the capacity to fulfill one’s role as a leader. This approach neglects more complex development categories at the group level [23]. 

Although only one study explicitly categorizes this focus [171], the documented interventions almost exclusively concentrate on leader development. This is because the development of individual capabilities of leaders is central to their approach. Aside from one study [32,79], followers are not considered in study designs. Therefore, art-based leader development, as reflected in evidence, aligns with the adult development paradigm. Art-based leader development is interpreted through concepts of identity and linked to skills of self-awareness, metacognition, and self-regulation. While supporting leaders’ mastery of cognitive, emotional, and interpersonal regulation, art-based methods currently embed leader development in adult development [203,204,205].

The studies on leader development corroborate evidence from reviews in other fields of adult development. Art-based methods have been shown to facilitate perceptual refinement [206,207] and reflective practice [208,209]. Furthermore, these methods have been shown to positively impact learners’ cognitive and emotional development [209,210]. This includes enhancing empathy [211], higher-order cognitive skills [212], and sense-making [213]. Moreover, the arts have been demonstrated to enhance group cohesion and interpersonal skills [209,214,215]. Additionally, art-based approaches have been shown to facilitate a shift in learners’ attitudes and to enhance their capacity to act [210]. It is noteworthy that research on art-based (leader) development does not identify any significant outcomes that are unique to leadership alone.

### 4.2. Paradigmatic Insights

Qualitative research is the dominant paradigm in the field. Qualitative studies yielded more significant and nuanced findings than quantitative studies, which were more limited in scope. Qualitative studies show considerable variability in terms of methodological rigor and reporting quality. Qualitative studies of lower quality are generally less robust in their evidence. Nevertheless, a synthesis of the evidence reveals a pattern of findings that are supported by studies with predominantly good to very good methodological quality, despite some shortcomings in reporting quality. A paucity of longitudinal studies exists in the research area, and only one study is sufficiently robust to demonstrate actual behavioral change resulting from a long-term leadership program [32,79].

The preference of researchers for qualitative study designs is also evident in other areas of art-based learning [121,215]. This can be explained, among other things, by the epistemological foundations of art-based methods. Art-based leadership development falls under constructivist learning interventions. These create a learning environment where participants can have experiences without clearly defined learning objectives [216]. Learner-centeredness and the reflection on experiences play an important role in this process. From this theoretical perspective, learning success in leadership development is seen in a deeper understanding of self and environment, enhanced self-reflection, and expanded problem-solving skills [217].

Qualitative methods are particularly adept at capturing complex, subjective experiences and meanings, inherently aligning with a constructivist paradigm [218]. The constructivist view, which posits that individuals interpret experiences and construct knowledge, strongly supports the qualitative investigation of interventions rooted in the same epistemological principles.

Another reason for the paucity of quantitative studies is the distinctive nature of arts-based practices. The learner-centered approach poses a challenge to the standardization of interventions and replication [219]. Comparative studies may encounter difficulties in drawing generalizable conclusions due to variability in implementation fidelity, instructor expertise, and student engagement, which can introduce heterogeneity. Furthermore, contextual variables may interact with the intervention, making it challenging to isolate the effects of arts-based practices [220].

The dominance of qualitative research, which inherently focuses on the singularity and unique characteristics of individual cases, leads to a diverse range of outcomes in art-based leadership development. In contrast, quantitative research typically focuses on a limited number of variables to accommodate a larger number of cases [131], which is reflected in intensity effect sizes. Apart from obstacles in the learning process, qualitative studies, unlike quantitative ones, exclusively report positive effects. This phenomenon appears to be typical for qualitative research on art-based methods [121]. It thus raises the question of whether there is a reporting bias in qualitative research on art-based leadership development, which may undermine the robustness of the findings.

### 4.3. Implications for Leadership Development

The mindsets of effective leadership prioritize learning and promote engaging relationships [221]. This points to reflexivity [12,222] and sense-making as essential leadership competencies [223,224]. Identity, meta-cognitive processes, and emotional regulation are considered key factors in developing leadership expertise [30]. Art-based leader development demonstrably fosters reflexivity, emotional transformation, higher-order cognitive skills, and sense-making. The reported effects are considered intrapersonal developmental indicators, which are believed to positively influence leader competence and effectiveness in the long term [26].

The findings indicate that art-based practices offer a promising approach to leader development, providing a holistic and transformative learning experience. The distinctive potential of art-based leader development lies in its capacity to alter leaders’ prevailing mindset. In this regard, art-based methods present distinctive opportunities for the development of critical leadership competencies that are not as readily addressed through traditional approaches, which are generally oriented towards skillsets, standardized, and performance-driven [1,221]. As a leadership development practice that incorporates critical reflection, art-based andragogy addresses many of the shortcomings identified in traditional approaches by promoting a deeper, more individualized learning process [225].

Although effective leadership necessitates crucial competencies such as direction setting and external environment navigation [26], art-based approaches have thus far scarcely addressed the strategic level of leadership skill requirements, such as decision-making and problem-solving, which are exceptionally important at the highest leadership levels [226]. There are no generalizable empirical results in this area. Art-based methods currently align more with relational and authentic leader capacities and underlying leadership theories than with strategic capabilities [205].

The existing research and practice in the field of art-based leader development both exhibit a failure to recognize that artistic practice is a creative and relational process. This process is characterized by deliberate uncertainty, which involves probing and shaping reality [227,228,229,230,231]. There is a paucity of research on the subject of negative capability and the capacity to think in the present moment when dealing with ambiguity and uncertainty, and developing creative solutions for complex problems, despite these being critical requirements for leaders [201,232,233,234]. In art-based leader development practice, sensory perceptiveness and awareness appear to play a minimal role, despite being fundamental to effective leadership [76,235,236]. It is noteworthy that art-based methods designed to enhance these competencies have been developed and empirically validated for efficacy in healthcare settings [207,237,238].

Empirical research on leadership development has demonstrated that art-based approaches may be superior to conventional programs in certain outcomes. However, other research has cast doubt on the superiority of arts-based approaches as a training resource [239]. This underscores the necessity for the development of both short-term interventions and long-term programs that are specifically designed to integrate the potential of aesthetic education and artistic processes with leadership theories and practical leadership requirements.

### 4.4. Limitations and Future Research Directions

This review provides a comprehensive overview of the evidence supporting the use of art-based methods in leadership development. It integrates different types of studies in terms of methodologies, recognizes the quality of the studies, and considers all types of evidence in the evidence synthesis. The assessment is limited to studies published in the English language and does not cover research beyond peer-reviewed academic journals. Despite the researchers’ best efforts to reduce bias, decisions that influence the outcome of the review—such as the selection of reports, quality assessment, and the construction of the thematic hierarchy—are ultimately based on the subjective judgment of the researchers [131].

On average, the qualitative studies in the sample exhibited satisfactory methodological rigor. In the conventional evidence hierarchy, which is often depicted as a pyramid, qualitative studies are often considered to be inferior to experimental study designs for demonstrating the effectiveness of interventions [240,241]. Nevertheless, more recent approaches in educational sciences support the concept of evidence-based practice using an evidence funnel, which considers the quantity and consistency across the body of evidence, thereby providing a more holistic view [242]. From this perspective, research on art-based leadership development provides a solid foundation for assessing what works.

Those who advocate for qualitative research on art-based methods view leadership development as a matter of personal growth and development. The ideal involves personal experience and humanistic values as a prerequisite for holistic transformation [22,89]. In contrast, the empiricist perspective places a premium on leaders’ behavior and performance, with an emphasis on the verifiability of development (see, for instance, [32,41]).

The central concepts of qualitative impact research, including reflective capacity, empathy, and interpersonal competencies, can be measured using validated scales and established inventories in psychology. This also applies to mindset as a psychological construct. These measurement instruments provide a foundation for future interdisciplinary research that could explore the complex, dynamic nature of leadership and the effects of art-based methods [1]. The development and psychometric testing of measurement instruments that account for the unique characteristics of art-based approaches represents a distinct research task [243].

The results of studies with follow-up assessments indicated that the impact of art-based leadership development can extend beyond the intervention period. However, there is a lack of research on the long-term effects of leadership development, both for conventional approaches [202] and art-based methods. Similarly, other application areas of art-based learning also document the absence of evidence on long-term impact [126]. Although outcomes that are considered indicators of long-term leadership development suggest that participants build and implement capacity over time [26], this has not been conclusively demonstrated. The role of time in leadership development represents a critical area for future research [202].

There is a notable divergence between the self-perception and the external perception of leaders. Leaders tend to evaluate their learning process and behavior more positively than their followers do [79]. Moreover, the methods commonly employed in art-based interventions, such as interviews, are vulnerable to the risk of social desirability bias [211]. Irrespective of the level of evidence, such discrepancies provide a compelling argument for the use of control group designs and supporting measures such as self-administered questionnaires and direct measurement [244,245]. Multi-group experimental designs allow for a comparison of the effectiveness of art-based and traditional leadership development.

In addition to the aforementioned neglected competencies, the differential impact of short-term and long-term interventions, particularly in terms of their transfer to professional practice, warrants further investigation [179]. A multitude of aspects pertaining to the design of art-based interventions in leadership development remain uncharted territory, including the influence of participant demographics (students vs. professionals) and facilitators. Moreover, the specific elements of art-based approaches that present challenges for learners remain under-researched. The reasons why interventions are not equally effective for everyone need to be explored. Furthermore, it is important to understand under what conditions the “constructive disturbance” [89] (p. 22) provokes a defensive attitude in learners that may prevent them from achieving learning goals.

In the field of conventional leadership development, there is a paucity of empirical research on the outcomes of collective initiatives [202]. In the context of an emerging intertwining of leadership development and a transformation of organizational culture, there is a necessity to expand both development initiatives and research efforts beyond individual development [38]. Although there is research indicating the positive impact of art-based interventions on organizational development and leadership, the evidence is not yet quantified [246]. This opens up a fundamental research avenue on the arts and leadership in social transformation.

## 5. Conclusions

This systematic review and qualitative metasummary have demonstrated the considerable impact of art-based leadership development on various dimensions of leadership capabilities. The findings indicate that art-based methods facilitate reflective and reflexive practices, higher-order cognitive skills, and emotional intelligence among participants. These methods also foster interpersonal competencies such as empathy and communication, which may contribute to a holistic transformation in leadership practice. Despite the promising results, the evidence predominantly focuses on individual development rather than collective outcomes within organizational contexts. The studies reviewed indicate a notable deficiency in robust empirical evidence, particularly concerning long-term effects.

In conclusion, art-based leadership development offers a comprehensive approach to changing leaders’ mindsets, enriching traditional leadership training paradigms. However, the field requires more rigorous, quantitative, and longitudinal research to substantiate these findings and explore the broader implications for relational leadership. Ultimately, the integration of artistic methods into leadership development programs holds significant potential for fostering transformative growth and enhancing leadership efficacy in complex and dynamic environments.

## Figures and Tables

**Table 3:** Exemplary thematic hierarchy.

Main Theme	Theme	Sub-Theme
Higher-order cognitive skills (14)	Reflective thinking (5)	Increased reflective thinking (2)
		Improved reflective thinking (1)
		Reflective thinking (1)
		Enhanced reflective learning (1)
	Critical thinking skills (3)	Improved critical thinking skills (1)
		Improved critical thinking (1)
		Improved problem-solving skills (1)
	Creativity (6)	Increased creativity (3)
		Reconnection with creativity (1)
		Enhanced creativity (1)
		Utilization of creative processes (1)

Note. Numbers in brackets indicate the number of reports contributing to a theme.

**Table 4:** Frequency effect sizes.

*Main Theme* Theme	No. of Studies (%)	Study (Quality Score/39)
*1 Learner engagement and satisfaction*		
Engagement	5 (15)	Andenoro & Ward (2008) [166] (17), Cranston & Kusanovich (2014) [168] (12), Rajendran & Andrew (2014) [51] (16), Singh & Widén (2020) [182] (13), Sutherland & Jelinek (2015) [22] (28)
Aesthetic experience	5 (15)	Harz et al. (2023) [172] (31), Parush & Koivunen (2014) [68] (17), Sandberg et al. (2023) [181] (31), Sutherland (2012) [183] (22), Sutherland & Jelinek (2015) [22] (28)
Satisfaction	5 (15)	Andenoro & Ward (2008) [166] (17), Cranston & Kusanovich (2013) [167] (12), Cranston & Kusanovich (2014) [168] (12), Harz et al. (2023) [172] (31), Hurdle & Greenhaw (2023) [173] (28)
*2 Learning process*		
Transformative learning	7 (21)	Firing et al. (2022) [171] (24), Hurdle & Greenhaw (2023) [173] (28), Parush & Koivunen (2014) [68] (17), Rajendran & Andrew (2014) [51] (16), Sutherland (2012) [183] (22), Winther & Højlund Larsen (2022) [185] (17), Woods et al. (2023) [45] (28)
Comprehensive learning	1 (3)	Rajendran & Andrew (2014) [51] (16)
*3 Sensory and experiential awareness*		
Sensitivity	7 (21)	Harz et al. (2023) [172] (31), Katz-Buonincontro (2011) [50] (10), Katz-Buonincontro & Phillips (2011) [176] (20), Katz-Buonincontro et al. (2015) [177] (11), Sandberg et al. (2023) [181] (31), Winther (2018) [184] (13), Woods et al. (2023) [45] (28)
*4 Emotional development and personal growth*		
Emotional awareness and transformation	10 (33)	Dennis (2014) [169] (6), Feltham (2012) [170] (10), Firing et al. (2022) [171] (24), Garavan et al. (2015) [69] (26), Hirsch et al. (2023) [57] (14), Katz-Buonincontro (2011) [50] (10), Leonard et al. (2013) [106] (22), Munro et al. (2015) [179] (18), Sutherland (2012) [183] (22), Winther & Højlund Larsen (2022) [185] (17)
Self-awareness	10 (30)	Dennis (2014) [169] (6), Hirsch et al. (2023) [57] (14), Munro et al. (2015) [179] (18), Peña & Grant (2019) [180] (20), Romanowska et al. (2014) [79] (34), Schyns et al. (2013) [35] (23), Sutherland (2012) [183] (22), Winther (2018) [184] (13), Winther & Højlund Larsen (2022) [185] (17), Woods et al. (2023) [45] (28)
Empowered self-concept	9 (27)	Dennis (2014) [169] (6), Feltham (2012) [170] (10), Kaimal et al. (2014) [174] (20), Kaimal et al. (2016) [175] (19), Parush & Koivunen (2014) [68] (17), Peña & Grant (2019) [180] (20), Romanowska et al. (2011) [67] (31), Winther (2018) [184] (13), Woods et al. (2023) [45] (28)
*5 Reflective and reflexive practices*		
Reflective and reflexive practices	15 (45)	Dennis (2014) [169] (6), Feltham (2012) [170] (10), Harz et al. (2023) [172] (31), Hirsch et al. (2023) [57] (14), Kaimal et al. (2014) [174] (20), Kaimal et al. (2016) [175] (19), Katz-Buonincontro (2011) [50] (10), Katz-Buonincontro & Phillips (2011) [176] (20), Katz-Buonincontro et al. (2015) [177] (11), Kilic (2023) [40] (30), Schyns et al. (2013) [35] (23), Sutherland (2012) [183] (22), Sutherland & Jelinek (2015) [22] (28), Winther & Højlund Larsen (2022) [185] (17), Woods et al. (2023) [45] (28)
*6 Higher-order cognitive skills*		
Reflective thinking	4 (12)	Andenoro & Ward (2008) [166] (17), Cranston & Kusanovich (2013) [167] (12), Katz-Buonincontro (2011) [50] (10), Leonard et al. (2013) [106] (22)
Critical thinking skills	3 (9)	Andenoro & Ward (2008) [166] (17), Katz-Buonincontro & Phillips (2011) [176] (20), Singh & Widén (2020) [182] (13)
Creativity	6 (18)	Hirsch et al. (2023) [57] (14), Kaimal et al. (2014) [174] (20), Katz-Buonincontro (2011) [50] (10), Katz-Buonincontro & Phillips (2011) [176] (20), Katz-Buonincontro et al. (2015) [177] (11), Kilic (2023) [40] (30)
*7 Sense-making*		
Sense-making	8 (24)	Dennis (2014) [169] (6), Firing et al. (2022) [171] (24), Harz et al. (2023) [172] (31), Hirsch et al. (2023) [57] (14), Katz-Buonincontro et al. (2015) [177] (11), Peña & Grant (2019) [180] (20), Singh & Widén (2020) [182] (13), Sutherland & Jelinek (2015) [22] (28)
*8 Adaptive resilience*		
Negative capability	2 (6)	Firing et al. (2022) [171] (24), Hirsch et al. (2023) [57] (14)
Risk taking	5 (15)	Cranston & Kusanovich (2014) [168] (12), Katz-Buonincontro & Phillips (2011) [176] (20), Katz-Buonincontro et al. (2015) [177] (11), Leonard et al. (2013) [106] (22), Parush & Koivunen (2014) [68] (17)
Mental health	4 (13)	Feltham (2012) [170] (10), Harz et al. (2023) [172] (31), Kilic (2023) [40] (30), Romanowska et al. (2011) [67] (31), Romanowska et al. (2013) [32] (34), Romanowska et al. (2014) [79] (34)
*9 Interpersonal and social competencies*		
Empathy	7 (21)	Andenoro & Ward (2008) [166] (17), Cranston & Kusanovich (2013) [167] (12), Feltham (2012) [170] (10), Firing et al. (2022) [171] (24), Harz et al. (2023) [172] (31), Kaimal et al. (2016) [175] (19), Katz-Buonincontro (2011) [50] (10)
Ethical understanding	3 (9)	Cranston & Kusanovich (2013) [167] (12), Cranston & Kusanovich (2014) [168] (12), Medeiros et al. (2012) [178] (7)
Communication skills	6 (18)	Garavan et al. (2015) [69] (26), Kilic (2023) [40] (30), Medeiros et al. (2012) [178] (7), Munro et al. (2015) [179] (18), Sandberg et al. (2023) [181] (31), Winther & Højlund Larsen (2022) [185] (17)
Interpersonal skills	8 (24)	Dennis (2014) [169] (6), Feltham (2012) [170] (10), Firing et al. (2022) [171] (24), Kilic (2023) [40] (30), Romanowska et al. (2014) [79] (34), Sutherland & Jelinek (2015) [22] (28), Winther & Højlund Larsen (2022) [185] (17), Woods et al. (2023) [45] (28)
Collaboration	7 (21)	Cranston & Kusanovich (2014) [168] (12), Firing et al. (2022) [171] (24), Katz-Buonincontro (2011) [50] (10), Kilic (2023) [40] (30), Leonard et al. (2013) [106] (22), Medeiros et al. (2012) [178] (7), Woods et al. (2023) [45] (28)
*10 Comprehensive leadership development*		
Leadership perspective	5 (15)	Andenoro & Ward (2008) [166] (17), Kaimal et al. (2014) [174] (20), Kaimal et al. (2016) [175] (19), Singh & Widén (2020) [182] (13), Woods et al. (2023) [45] (28)
Leadership enhancement	4 (12)	Cranston & Kusanovich (2014) [168] (12), Garavan et al. (2015) [69] (26), Medeiros et al. (2012) [178] (7), Romanowska et al. (2014) [79] (34)
Embodied leadership	3 (9)	Sandberg et al. (2023) [181] (31), Winther (2018) [184] (13), Winther & Højlund Larsen (2022) [185] (17)
*11 Transfer success*		
Real-world application	9 (27)	Andenoro & Ward (2008) [166] (17), Cranston & Kusanovich (2013) [167] (12), Kaimal et al. (2014) [174] (20), Kaimal et al. (2016) [175] (19), Leonard et al. (2013) [106] (22), Sandberg et al. (2023) [181] (31), Singh & Widén (2020) [182] (13), Sutherland (2012) [183] (22), Sutherland & Jelinek (2015) [22] (28)
Behavioral change	2 (6)	Feltham (2012) [170] (10), Romanowska et al. (2013) [32] (34), Romanowska et al. (2014) [79] (34)

Note. No. = number.

**Table 5:** Intensity effect sizes.

Reference	No. of Themes with Frequency Effect Size ≥25	Intensity Score A (Column B/Total Themes with Frequency Effect Size ≥25 (5)) (%)	Total No. of Themes	Intensity Score B (Column D/Total Themes (27)) (%)	QuADS Score/39
Andenoro & Ward (2008) [166]	1	20	7	26	17
Cranston & Kusanovich (2013) [167]	1	20	5	19	12
Cranston & Kusanovich (2014) [168]	0	0	6	22	12
Dennis (2014) [169]	4	80	6	22	6
Feltham (2012) [170]	3	60	7	26	10
Firing et al. (2022) [171]	1	20	6	26	24
Garavan et al. (2015) [69]	1	20	3	11	26
Harz et al. (2023) [172]	1	20	7	26	31
Hirsch et al. (2023) [57]	3	60	6	22	14
Hurdle & Greenhaw (2023) [173]	0	0	2	7	28
Kaimal et al. (2014) [174]	3	60	5	19	20
Kaimal et al. (2016) [175]	3	60	5	19	19
Katz-Buonincontro (2011) [50]	2	40	7	26	10
Katz-Buonincontro & Phillips (2011) [176]	1	20	5	19	20
Katz-Buonincontro et al. (2015) [177]	1	20	5	19	11
Kilic (2023) [40]	1	20	5	22	30
Leonard et al. (2013) [106]	2	40	5	19	22
Medeiros et al. (2012) [178]	0	0	4	15	7
Munro et al. (2015) [179]	2	40	3	11	18
Parush & Koivunen (2014) [68]	1	20	4	15	17
Peña & Grant (2019) [180]	2	40	3	11	20
Rajendran & Andrew (2014) [51]	0	0	3	11	16
Romanowska et al. (2011) [67]	1	20	2	7	31
Romanowska et al. (2013) [32]	0	0	2	7	34
Romanowska et al. (2014) [79]	1	20	5	19	34
Sandberg et al. (2023) [181]	1	20	5	19	31
Schyns et al. (2013) [35]	2	40	2	7	23
Singh & Widén (2020) [182]	1	20	5	19	13
Sutherland (2012) [183]	4	80	6	22	22
Sutherland & Jelinek (2015) [22]	2	40	6	22	28
Winther (2018) [184]	2	40	4	15	17
Winther & Højlund Larsen (2022) [185]	3	60	7	26	13
Woods et al. (2023) [45]	3	60	8	30	28

Note. No. = number.

**Table 6:** Study dynamics.

Author(s)	Intervention Frequency	Participant Type	Study Design	Sampling	Study Group Design	Data Collection Timepoint	Post-Intervention Data Collection Interval	Assessment Tool	Quality Score/39
Andenoro & Ward (2008) [166]	Multiple sessions over 15 weeks	Enrolled students	Case study	Purposive	N/A	During intervention Post-intervention	N/A End of intervention	N/A	17
Cranston & Kusanovich (2013) [167]	Single 2-day session	Not reported	Qualitative descriptive study	Not reported	N/A	Pre-intervention During intervention Post-intervention	Not reported N/A Not reported	N/A	12
Cranston & Kusanovich (2014) [168]	Single 2-day session	Workshop attendees	Qualitative descriptive study	Convenience	N/A	During intervention	N/A	N/A	12
Dennis (2014) [169]	Single 2-day sessions	Not reported	Qualitative action research study	Not reported	N/A	Not reported	Not reported	N/A	6
Feltham (2012) [170]	2 separate one-day sessions	Individuals connected with the training event	Qualitative descriptive study	Purposive	N/A	Not reported	Not reported	N/A	10
Firing et al. (2022) [171]	9-week program	Enrolled students	Case study	Purposive	N/A	During intervention (observation) Not reported (interviews)	N/A Not reported	N/A	24
Garavan et al. (2015) [69]	Single session	Leaders from a large company	Quasi-experimental study	Random	Comparative trial	Pre-test Post-test	6 months	Subjective rating scales	26
Harz et al. (2023) [172]	Single one and a half day session	Enrolled students	Qualitative descriptive study	Purposive	N/A	Post-inter- vention	End of intervention	N/A	31
Hirsch et al. (2023) [57]	Single session	Not reported	Qualitative descriptive study	Not reported	N/A	Post-inter- vention	Six-week period	N/A	14
Hurdle & Greenhaw (2023) [173]	2 sessions one week apart	Enrolled students	Case Study	Purposive	N/A	Post-intervention	One week	N/A	28
Kaimal et al. (2014) [174]	3 separate sessions	Program participants	Case study	Purposive	N/A	Not reported	Not reported	N/A	20
Kaimal et al. (2016) [175]	2 separate sessions	Program participants	Case study	Purposive	N/A	During intervention Post-intervention	N/A Not reported	N/A	19
Katz-Buonincontro (2011) [50]	5-day program	Program participants	Case study	Purposive	N/A	During intervention	N/A	N/A	10
Katz-Buonincontro & Phillips (2011) [176]	11-week course 5-day program	Enrolled students Program participants	Comparative case study	Purposive	N/A	Not reported	N/A	N/A	20
Katz-Buonincontro et al. (2015) [177]	Not reported	Program participants	Comparative case study	Purposive	N/A	Not reported	N/A	N/A	11
Kilic (2023) [40]	Not reported	Program participants	Mixed-methods action research study Quasi-experimental sub-study	Purposive convenience	Single group design	Pre-test During intervention Post-test	Not reported	Social Skills Inventory (SSI) Affect Grid	30
Leonard et al. (2013) [106]	2-h session	Two unipro- fessional groups	Qualitative descriptive study	Purposive	N/A	Post-intervention	7 days 4 to 9 months	N/A	22
Medeiros et al. (2012) [178]	Single session	Enrolled students	Quantitative descriptive study	Purposive	N/A	Post-test	Not reported	Subjective rating scales	7
Munro et al. (2015) [179]	Intermittent sessions over 18-month period	Company members	Quantitative exploratory study	Self-selection	Single group design	Pre-test Post-test	Not reported	Bar-On Emotional Quotient Inventory (EQi) Neethling Brain Instrument® (NBI)	18
Parush & Koivunen (2014) [68]	Single sessions	Program participants Company members	Case study	Convenience	N/A	During intervention Post-intervention	N/A Not reported	N/A	17
Peña & Grant (2019) [180]	2-h session	Enrolled students	Qualitative phenomenological study	Self-selection	N/A	Post-intervention	Not reported	N/A	20
Rajendran & Andrew (2014) [51]	Single session	Enrolled students	Qualitative action research study	Convenience	N/A	Post-intervention	Not reported	N/A	16
Romanowska et al. (2011) [67]	12 intermittent 3-h sessions over one year	Managers and their subordinates	Experimental study	Random	Comparative trial	Pre-test Post-test	12 months 18 months	Maslach Burnout Inventory Karolinska Sleep Questionnaire Hopkins Symptom Checklist Covert Coping Index Biometric instruments	31
Romanowska et al. (2013) [32]	12 intermittent 3-h sessions over one year	Managers and their subordinates	Experimental study	Random	Comparative trial	Pre-test Post-test	12 months 18 months	Developmental Leadership Questionnaire (DLQ) NEO-PI-R Sense of Coherence questionnaire (SOC)	34
Romanowska et al. (2014) [79]	12 intermittent 3-h sessions over one year	Managers and their subordinates	Experimental study	Random	Comparative trial	Pre-test Post-test	12 months 18 months	Developmental Leadership Questionnaire (DLQ)	34
Sandberg et al. (2023) [181]	2-day sessions	Managers	Mixed- methods study Quasi-experimental sub-study	Self-selection	Single group design	Pre-test Post-test	Up to 2 weeks and 6 weeks (questionnaire) 13 to 17 weeks (interviews)	Subjective rating scales	31
Schyns et al. (2013) [35]	Single session	Enrolled students	Qualitative descriptive study	Convenience	N/A	During intervention	N/A	N/A	23
Singh & Widén (2020) [182]	Single session	Enrolled students	Qualitative descriptive study	Convenience	N/A	Post-intervention	Not reported	N/A	13
Sutherland (2012) [183]	1-day session	Enrolled students	Qualitative grounded theory study	Convenience	N/A	Post-intervention	Not reported	N/A	22
Sutherland & Jelinek (2015) [22]	1-day session	Enrolled students	Case study	Convenience	N/A	Post-intervention	24 h 6 to 12 months	N/A	28
Winther (2018) [184]	Not reported	Enrolled students	Qualitative performative study	Convenience	N/A	Not reported	N/A	N/A	13
Winther & Højlund Larsen (2022) [185]	3-month course	Program participants	Qualitative phenomenological study	Convenience	N/A	During intervention	N/A	N/A	17
Woods et al. (2023) [45]	2-h session	Workshop attendees	Qualitative action research study	Self-selection	N/A	During intervention Post-intervention	N/A Not reported	N/A	28
Andenoro & Ward (2008) [166]	Multiple sessions over 15 weeks	Enrolled students	Case study	Purposive	N/A	During intervention Post-intervention	N/A End of intervention	N/A	17
Cranston & Kusanovich (2013) [167]	Single 2-day session	Not reported	Qualitative descriptive study	Not reported	N/A	Pre-intervention During intervention Post-intervention	Not reported N/A Not reported	N/A	12
Cranston & Kusanovich (2014) [168]	Single 2-day session	Workshop attendees	Qualitative descriptive study	Convenience	N/A	During intervention	N/A	N/A	12
Dennis (2014) [169]	Single 2-day sessions	Not reported	Qualitative action research study	Not reported	N/A	Not reported	Not reported	N/A	6
Feltham (2012) [170]	2 separate one-day sessions	Individuals connected with the training event	Qualitative descriptive study	Purposive	N/A	Not reported	Not reported	N/A	10
Firing et al. (2022) [171]	9-week program	Enrolled students	Case study	Purposive	N/A	During intervention (observation) Not reported (interviews)	N/A Not reported	N/A	24
Garavan et al. (2015) [69]	Single session	Leaders from a large company	Quasi-experimental study	Random	Comparative trial	Pre-test Post-test	6 months	Subjective rating scales	26
Harz et al. (2023) [172]	Single one and a half day session	Enrolled students	Qualitative descriptive study	Purposive	N/A	Post-intervention	End of intervention	N/A	31
Hirsch et al. (2023) [57]	Single session	Not reported	Qualitative descriptive study	Not reported	N/A	Post-intervention	Six-week period	N/A	14
Hurdle & Greenhaw (2023) [173]	2 sessions one week apart	Enrolled students	Case Study	Purposive	N/A	Post-intervention	One week	N/A	28
Kaimal et al. (2014) [174]	3 separate sessions	Program participants	Case study	Purposive	N/A	Not reported	Not reported	N/A	20
Kaimal et al. (2016) [175]	2 separate sessions	Program participants	Case study	Purposive	N/A	During intervention Post-intervention	N/A Not reported	N/A	19
Katz-Buonincontro (2011) [50]	5-day program	Program participants	Case study	Purposive	N/A	During intervention	N/A	N/A	10
Katz-Buonincontro & Phillips (2011) [176]	11-week course 5-day program	Enrolled students Program participants	Comparative case study	Purposive	N/A	Not reported	N/A	N/A	20
Katz-Buonincontro et al. (2015) [177]	Not reported	Program participants	Comparative case study	Purposive	N/A	Not reported	N/A	N/A	11
Kilic (2023) [40]	Not reported	Program participants	Mixed-methods action research study Quasi-experimental sub-study	Purposive convenience	Single group design	Pre-test During intervention Post-test	Not reported	Social Skills Inventory (SSI) Affect Grid	30
Leonard et al. (2013) [106]	2 h session	Two uni-professional groups	Qualitative descriptive study	Purposive	N/A	Post-intervention	7 days 4 to 9 months	N/A	22
Medeiros et al. (2012) [178]	Single session	Enrolled students	Quantitative descriptive study	Purposive	N/A	Post-test	Not reported	Subjective rating scales	7
Munro et al. (2015) [179]	Intermittent sessions over 18-month period	Company members	Quantitative exploratory study	Self-selection	Single group design	Pre-test Post-test	Not reported	Bar-On Emotional Quotient Inventory (EQi) Neethling Brain Instrument® (NBI)	18
Parush & Koivunen (2014) [68]	Single sessions	Program participants Company members	Case study	Convenience	N/A	During intervention Post-intervention	N/A Not reported	N/A	17
Peña & Grant (2019) [180]	2 h session	Enrolled students	Qualitative phenomenological study	Self-selection	N/A	Post-intervention	Not reported	N/A	20
Rajendran & Andrew (2014) [51]	Single session	Enrolled students	Qualitative action research study	Convenience	N/A	Post-intervention	Not reported	N/A	16
Romanowska et al. (2011) [67]	12 intermittent 3 h sessions over one year	Managers and their subordinates	Experimental study	Random	Comparative trial	Pre-test Post-test	12 months 18 months	Maslach Burnout Inventory Karolinska Sleep Questionnaire Hopkins Symptom Checklist Covert Coping Index Biometric instruments	31
Romanowska et al. (2013) [32]	12 intermittent 3 h sessions over one year	Managers and their subordinates	Experimental study	Random	Comparative trial	Pre-test Post-test	12 months 18 months	Developmental Leadership Questionnaire (DLQ) NEO-PI-R Sense of Coherence questionnaire (SOC)	34
Romanowska et al. (2014) [79]	12 intermittent 3 h sessions over one year	Managers and their subordinates	Experimental study	Random	Comparative trial	Pre-test Post-test	12 months 18 months	Developmental Leadership Questionnaire (DLQ)	34
Sandberg et al. (2023) [181]	2-day sessions	Managers	Mixed-methods study Quasi-experimental sub-study	Self-selection	Single group design	Pre-test Post-test	Up to 2 weeks and 6 weeks (questionnaire) 13 to 17 weeks (interviews)	Subjective rating scales	31
Schyns et al. (2013) [35]	Single session	Enrolled students	Qualitative descriptive study	Convenience	N/A	During intervention	N/A	N/A	23
Singh & Widén (2020) [182]	Single session	Enrolled students	Qualitative descriptive study	Convenience	N/A	Post-intervention	Not reported	N/A	13
Sutherland (2012) [183]	1-day session	Enrolled students	Qualitative grounded theory study	Convenience	N/A	Post-intervention	Not reported	N/A	22
Sutherland & Jelinek (2015) [22]	1-day session	Enrolled students	Case study	Convenience	N/A	Post-intervention	24 h 6 to 12 months	N/A	28
Winther (2018) [184]	Not reported	Enrolled students	Qualitative performative study	Convenience	N/A	Not reported	N/A	N/A	13
Winther & Højlund Larsen (2022) [185]	3-month course	Program participants	Qualitative phenomenological study	Convenience	N/A	During intervention	N/A	N/A	17
Woods et al. (2023) [45]	2 h session	Workshop attendees	Qualitative action research study	Self-selection	N/A	During intervention Post-intervention	N/A Not reported	N/A	28

Note. N/A = not applicable.

**Table 7:** QuADS mean scores.

Item	Mean Score/3
1 Theoretical or conceptual underpinning to the research	1.8
2 Statement of research aim/s	2.0
3 Clear description of research setting and target population	2.5
4 The study design is appropriate to address the stated research aim/s	2.2
5 Appropriate sampling to address the research aim/s	0.8
6 Rationale for choice of data collection tool/s	1.1
7 The format and content of data collection tool is appropriate to address the stated research aim/s	2.2
8 Description of data collection procedure	1.6
9 Recruitment data provided	1.3
10 Justification for analytic method selected	1.0
11 The method of analysis was appropriate to answer the research aim/s	2.2
12 Evidence that the research stakeholders have been considered in research design or conduct	0.2
13 Strengths and limitations critically discussed	1.0
Total score	20/39

## Data Availability

The original contributions presented in the study are included in the article/Supplementary Materials, further inquiries can be directed to the author.

## Supplementary Materials

The following supporting information can be downloaded at: https://www.mdpi.com/article/10.3390/bs14080714/s1, Table S1: Search strategy; Table S2: Thematic hierarchy; Code descriptions: Outcome summary.
