# Supplementary material for: Outcomes of Art-Based Leadership Development: A Qualitative Metasummary
Source: Behav Sci (Basel). 2024 Aug 14;14(8):714. doi: 10.3390/bs14080714 (PMC11351991; doi:10.3390/bs14080714)
Supplement: Supplementary file 1 [file behavsci-14-00714-s001.zip › Supplement 3_Outcome Summary.pdf]

Andenoro & Ward (2008) [166]

Summary of Empirical Results and Effects of Art-Based Pedagogy from Andenoro & Ward (2008) [166]

Empirical Results:

The study found that the use of popular culture and films as an instructional strategy in leadership education had significant positive effects on participants. Specifically:

1. Increased Engagement: Participants reported that the course format, which included the use of films, was more engaging compared to traditional lecture-based courses. They appreciated the direct application of leadership theories seen in movies and felt this approach made learning more enjoyable and relatable.
2. Enhanced Critical Thinking Skills: The study aimed to develop six core critical thinking skills: interpretation, analysis, evaluation, inference, explanation, and self-regulation. Participants noted improvement in these skills through their engagement with the course materials and activities. They found themselves interpreting, analyzing, and evaluating leadership roles and decisions depicted in films, which influenced their understanding and application of leadership principles.
3. Broader Perspective: Participants indicated that watching films with a leadership focus allowed them to see leadership in action from various perspectives. This broadened their understanding and appreciation of different leadership styles and approaches.
4. Real-World Application: The study showed that the art-based approach helped students apply leadership theories to real-world situations. The use of films provided concrete examples of leadership, making theoretical concepts more tangible and easier to grasp.
5. Reflective Thinking: The inclusion of reflective evaluations and discussions was beneficial in helping students synthesize their learning and apply it to their own experiences. This reflective practice was crucial in developing deeper insights into leadership and critical thinking.

Effects of Art-Based Approach:

- Enhanced Empathy: Students could empathize with characters and their leadership challenges, fostering a deeper understanding of leadership dynamics.
- Increased Reflectivity: Reflective evaluations and discussions promoted self-examination and self-correction among participants, enhancing their ability to think critically about their actions and decisions.
- High Satisfaction with Course Design: The innovative use of films and popular culture was well-received, with students expressing high satisfaction with this non-traditional approach to learning.

Keywords for Data Summary Table

- Enhanced engagement
- Improved critical thinking skills
- Broader leadership perspective
- Real-world application of theories
- Increased reflective thinking
- Enhanced empathy
- High satisfaction with course design

These keywords reflect the main outcomes of the study as it pertains to the effects of the art-based approach on participants.

Cranston & Kusanovich (2013) [167]

Summary of Empirical Results and Effects of Art-Based Pedagogy from Cranston & Kusanovich (2013) [167]

Empirical Results:

1. Enhanced Understanding of Ethical Dimensions:

Participants reported a deeper understanding of the ethical dimensions of decision-making in educational leadership.

The dramatized scenarios allowed participants to engage with ethical dilemmas more profoundly than traditional methods.

2. Increased Empathy:

The use of dramatic arts fostered a greater sense of empathy among participants.

Participants could better appreciate the perspectives and emotional experiences of others involved in ethical dilemmas.

3. Improved Reflective Thinking:

The art-based approach encouraged participants to reflect more deeply on their own values and ethical decision-making processes.

The workshop facilitated a space for participants to consider multiple perspectives and outcomes.

4. Enhanced Engagement and Enjoyment:

Participants found the art-based approach more engaging and enjoyable compared to traditional case study methods.

The interactive and embodied nature of the workshops made the learning experience more dynamic and memorable.

5. Development of Practical Skills:

Participants noted improvements in their ability to handle complex, real-world ethical dilemmas.

The role-playing and dramatization helped in practicing and internalizing leadership skills in a safe, controlled environment.

Effects of Art-Based Approach:

- Enhanced Empathy: Participants developed a greater ability to empathize with others, understanding the emotional and ethical complexities involved in leadership decisions.
- Increased Reflectivity: The approach promoted reflective thinking, encouraging participants to analyze and evaluate their decision-making processes more critically.
- High Satisfaction with Course Design: Participants expressed high satisfaction with the interactive and engaging nature of the course.
- Improved Ethical Understanding: The dramatized scenarios helped participants to better grasp the ethical implications of their decisions.
- Real-World Application: The skills and insights gained from the workshops were directly applicable to participants' professional roles, enhancing their practical leadership capabilities.

Keywords for Data Summary Table

- Enhanced empathy
- Increased reflective thinking
- High satisfaction with course design
- Improved ethical understanding
- Real-world application

These keywords reflect the main outcomes of the study, highlighting the positive impacts of an art-based approach to leadership education.

Cranston & Kusanovich (2014) [168]

Summary of Empirical Results and Effects of Art-Based Pedagogy from Cranston & Kusanovich (2014) [168]

Empirical Results:

1. Enhanced Ethical Understanding:

Participants developed a deeper, embodied understanding of the ethical dimensions of decision-making in educational leadership.

The dramatized scenarios helped participants experience ethical dilemmas in a tangible, impactful way.

2. Increased Empathy and Perspective-Taking:

Participants reported a greater ability to empathize with others and appreciate multiple perspectives, particularly those of various stakeholders in educational settings.

3. Improved Reflective Thinking:

The use of applied drama encouraged participants to reflect more deeply on their own values, decision-making processes, and the complexities of ethical leadership.

4. Greater Engagement and Enjoyment:

Participants found the art-based approach more engaging and enjoyable compared to traditional methods.

The interactive nature of the workshops made learning more dynamic and memorable.

5. Development of Practical Skills:

Participants noted improvements in their ability to handle real-world ethical dilemmas.

The role-playing and dramatization helped internalize leadership skills and ethical frameworks.

6. Enhanced Collaborative Decision-Making:

Participants emphasized collaborative and consultative decision-making processes.

The workshops fostered a sense of team-building and collective problem-solving.

Effects of Art-Based Approach:

- Enhanced Empathy: Participants developed greater empathy for others, understanding the emotional and ethical complexities of leadership decisions.
- Increased Reflectivity: Reflective thinking was promoted, encouraging participants to critically analyze their decision-making processes.
- High Satisfaction with Course Design: The innovative and engaging nature of the course was well-received, leading to high satisfaction.
- Improved Ethical Understanding: The dramatized scenarios helped participants better grasp the ethical implications of their decisions.
- Real-World Application: The skills and insights gained from the workshops were directly applicable to professional roles, enhancing practical leadership capabilities.
- Enhanced Collaborative Decision-Making: Participants reported an increased ability to engage in collaborative decision-making, valuing the input and perspectives of various stakeholders.

Keywords for Data Summary Table

- Enhanced ethical understanding
- Increased empathy
- Improved reflective thinking
- Greater engagement
- Development of practical skills
- Enhanced collaborative decision-making
- High satisfaction with course design

These keywords reflect the main outcomes of the study, highlighting the positive impacts of an art-based approach to leadership education.

"The third theme to emerge was the perception of the relevance of personal risk-taking within a group setting to the overall learning process."
"Several participants seemed to be referring to some sort of risk-taking or challenge that was required of them because of the unfamiliar methods."
"This finding, that there was some awareness of the risk taking involved in the performing arts process, seemed to be acknowledged in numerous ways throughout the journals."

- Relevance of risk-taking

Dennis (2014) [169]

Summary of Empirical Results and Effects of Art-Based Pedagogy from Dennis (2014) [169]

Empirical Results:

1. Incremental Exploration and Self-Awareness:

- The application of improvisation facilitated a deeper exploration into self-awareness and understanding of others.

- Participants engaged in physical, spatial, temporal, and imagistic structures derived from dance and drama, which required focus and self-discipline.

2. Enhanced Interpersonal Efficacy:

- Improvisation provided a framework for improving interpersonal efficacy in both individual artistry and team creativity.

- The nonverbal vocabulary and improvisation structures enabled participants to develop their repertoire incrementally, leading to better leadership skills.

3. Emotional Engagement and Transformation:

- Participants experienced an expansion of their expressive emotional palette, learning to translate negative emotions like anxiety and confusion into creative energy.

- The process enabled them to slow down their response rate and develop the capacity to choose their responses under pressure.

4. Development of Personal Agency and Self-Responsibility:

- The creative exchanges made personal agency and self-responsibility more visible and acknowledged by both self and others.

- Participants reported intangible positive outcomes, such as increased optimism, friendship, closeness, intimacy, and a feeling of energy or dynamism in the group.

5. Reflective and Dialogic Spaces:

- The method opened up reflective and dialogic spaces where participants could critically observe themselves and others, fostering a collaborative learning environment.

- The structures of improvised performance encouraged participants to reflect on their behavior and interactions, leading to personal and group development.

6. Cultural and Counter-Cultural Exploration:

- Improvised performance enabled exploration of cultural and counter-cultural interactions, fostering self-respect and respect for others.

- Participants engaged in exercises like maintaining soft eye contact, which facilitated increased mutual respect and coherence in teams.

Effects of Art-Based Approach:

- Enhanced Self-Awareness: Incremental exploration led to greater self-awareness and understanding of others.

- Improved Interpersonal Efficacy: Enhanced ability to lead, follow, and support within a team.

- Emotional Transformation: Translated negative emotions into creative energy, improving emotional management.

- Increased Personal Agency: Greater visibility and acknowledgment of personal agency and self-responsibility.

- Reflective Practice: Created spaces for critical reflection and dialogue, fostering collaborative learning.

- Cultural Exploration: Improved self-respect and respect for others through cultural and counter-cultural interactions.

Keywords for Data Summary Table

- Enhanced self-awareness
- Improved interpersonal efficacy
- Emotional transformation
- Increased personal agency
- Reflective practice
- Cultural exploration

These keywords reflect the main outcomes of the study, highlighting the positive impacts of an art-based approach to leadership development.

Feltham (2012) [170]

Summary of Empirical Results and Effects of Art-Based Pedagogy from Feltham (2012) [170]

Empirical Results:

1. Improved Interpersonal Skills:

- Participants developed significant improvements in interpersonal skills, crucial for effective management and collaboration.
- These skills included better communication, negotiation, and conflict resolution capabilities.

2. Enhanced Reflective Practice:

- The theatre-based approach fostered a high degree of reflexivity among participants.
- Participants reported increased self-awareness and the ability to critically evaluate their behaviors and leadership styles.

3. Increased Empathy and Emotional Intelligence:

- The program enhanced participants' empathy and emotional intelligence.
- Participants became more adept at understanding and managing their own emotions and those of others, leading to improved interpersonal relationships.

4. Behavioral Change and Confidence:

- Participants demonstrated notable behavioral changes, including increased confidence in handling difficult conversations and challenging situations.
- The safe environment provided by the actor-based role plays allowed participants to experiment with new behaviors and receive immediate feedback.

5. Stress Management and Well-being:

- Although the working environment remained stressful, participants felt better equipped to manage stress and reported a sense of improved well-being.
- The theatre-based training provided tools for coping with the pressures and uncertainties of their roles.

Effects of Art-Based Approach:

- Improved Interpersonal Skills: Enhanced communication, negotiation, and conflict resolution capabilities.
- Enhanced Reflective Practice: Increased self-awareness and critical evaluation of behaviors and leadership styles.
- Increased Empathy and Emotional Intelligence: Better understanding and management of emotions, leading to improved relationships.
- Behavioral Change and Confidence: Greater confidence and willingness to handle difficult conversations and situations.
- Stress Management and Well-being: Better equipped to manage stress and a sense of improved well-being.

Keywords for Data Summary Table

- Improved interpersonal skills
- Enhanced reflective practice
- Increased empathy
- Enhanced emotional intelligence
- Behavioral change
- Increased confidence
- Stress management
- Improved well-being

These keywords reflect the main outcomes of the study, highlighting the positive impacts of an art-based approach to leadership development.

Firing et al. (2022) [171]

Summary of Empirical Results and Effects of Art-Based Pedagogy from Firing et al. (2022) [171]

Empirical Results:

1. Transformative Learning:

- The participants reported significant transformative learning outcomes. This included a shift in their self-perception and a deeper understanding of their roles and responsibilities as leaders.

2. Holistic Identity Development:

- The theatre-based pedagogy facilitated the development of a holistic identity among participants. They experienced an integration of their personal and professional selves, enhancing their overall sense of identity.

3. Increased Empathy:

- The approach significantly increased empathy among participants. They developed a better understanding of themselves and others, which translated into more empathetic leadership practices.

4. Enhanced Emotional and Social Awareness:

- Participants showed enhanced emotional and social awareness. They became more attuned to their own emotions and those of others, leading to improved interpersonal interactions.

5. Community Building:

- The process fostered a strong sense of community among participants. They felt a greater connection with their peers, which contributed to a supportive and collaborative learning environment.

6. Overcoming Anxiety and Uncertainty:

- Participants learned to navigate and overcome feelings of uncertainty and anxiety. The theatre-based activities provided a safe space to explore and manage these emotions constructively.

Effects of Art-Based Approach:

- Transformative Learning: Significant shifts in self-perception and understanding of leadership roles.
- Holistic Identity Development: Integration of personal and professional identities.
- Increased Empathy: Better understanding of self and others, leading to empathetic leadership.
- Enhanced Emotional and Social Awareness: Improved emotional and social attunement.
- Community Building: Stronger sense of connection and support among participants.
- Overcoming Anxiety and Uncertainty: Constructive management of uncertainty and anxiety.

Keywords for Data Summary Table

- Transformative learning
- Holistic identity development
- Increased empathy
- Enhanced emotional awareness
- Enhanced social awareness
- Community building
- Overcoming anxiety
- Managing uncertainty

These keywords summarize the primary outcomes of the study, highlighting the positive impacts of an art-based approach to leadership development.

Garavan et al. (2015) [69]

Summary of Empirical Results and Effects of Art-Based Pedagogy from Garavan et al. (2015) [69]

Empirical Results:

1. Improved Emotional Intelligence:

Participants in the arts-based intervention demonstrated significant improvements in emotional intelligence compared to those in conventional interventions. The leadership drawing exercise helped participants develop better emotional awareness and control.

2. Enhanced Leader Identity:

The study showed that participants who engaged in the arts-based intervention experienced a stronger sense of leader identity. They felt more confident in their roles and developed a clearer vision of themselves as leaders.

3. Increased Feedback Orientation:

Participants exhibited a greater orientation towards seeking and utilizing feedback. The arts-based approach encouraged a more positive attitude towards feedback, enhancing participants' ability to process and act on feedback effectively.

4. No Significant Change in Openness to Experience:

While the arts-based intervention aimed to influence openness to experience, the results indicated no significant change in this dimension. Participants did not show a marked difference in their openness to new experiences compared to those in conventional interventions.

Effects of Art-Based Approach:

- Improved Emotional Intelligence: Participants developed a better understanding and management of their emotions, crucial for effective leadership.

- Enhanced Leader Identity: The intervention strengthened participants' identification with their leadership roles, boosting their confidence and clarity in their leadership capabilities.

- Increased Feedback Orientation: The arts-based approach fostered a more receptive and constructive attitude towards feedback, improving participants' ability to grow and adapt based on feedback.

Keywords for Data Summary Table

- Improved emotional intelligence
- Enhanced leader identity
- Increased feedback orientation

These keywords reflect the main outcomes of the study, highlighting the positive impacts of an art-based approach to leadership development.

Harz et al. (2023) [172]

Summary of Empirical Results and Effects of Art-Based Pedagogy from Harz et al. (2023) [172]

Empirical Results:

1. High Satisfaction with Course Design:

- The overall quality of the experience was rated very high, with a mean score of 4.86 out of 5, indicating strong participant satisfaction.

- The content was deemed current, accurate, interesting, and valuable, with mean scores around 4.8.

2. Enhanced Empathy and Emotional Engagement:

- The arts and humanities experience encouraged students to reflect on their professional roles and develop greater empathy.

- Participants reported an increased understanding of vulnerability and the importance of maintaining authenticity in patient interactions.

3. Improved Reflective Practice:

- Participants engaged in reflective practice, considering how their values and ideals impact their professional lives.

- The activity emphasized the importance of reflection in developing a compassionate and patient-centered approach to medicine.

4. Greater Appreciation for the Arts in Medical Education:

- The study underscored the significance of incorporating arts and humanities into medical training.

- Students expressed a renewed commitment to integrating these disciplines into their future practice, recognizing their role in fostering empathy, creativity, and holistic care.

Effects of Art-Based Approach:

- High Satisfaction with Course Design: Strong participant satisfaction and positive evaluation of the course content.

- Enhanced Empathy: Increased understanding and empathy towards patients.

- Improved Reflective Practice: Emphasis on reflection in professional development.

- Appreciation for the Arts: Recognized importance of arts in fostering a compassionate and creative approach to medicine.

Keywords for Data Summary Table

- High satisfaction with course design
- Enhanced empathy
- Improved reflective practice
- Appreciation for the arts

These keywords reflect the main outcomes of the study, highlighting the positive impacts of an art-based approach to medical education and leadership development.

“The codes ‘awareness’ and ‘broader perspective’ were applied 12 times each (6.7%) and related to students willing to work on being more observant and better listeners, while also considering different viewpoints.”

- Awareness
- Broader perspective

“The codes ‘reflection’ and ‘wellbeing’ were also considered as important changes participants were encouraged to consider after attending the AH session.”

- Wellbeing

Hirsch et al. (2023) [57]

Summary of Empirical Results and Effects of Art-Based Pedagogy from Hirsch et al. (2023) [57]

Empirical Results:

1. Development of Negative Capability:

- Participants learned to access and utilize Negative Capability, allowing them to remain present and open in situations of uncertainty and complexity.
- This capability enabled them to navigate professional challenges with a more reflective and less reactive approach.

2. Increased Self-Awareness and Emotional Regulation:

- Participants developed heightened self-awareness, recognizing their emotional and physical responses in stressful situations.
- Techniques such as deep breathing and mindfulness helped them manage their emotional reactions, leading to calmer and more measured responses.

3. Integration of Body and Mind:

- The study highlighted the importance of connecting mind and body, with participants learning to recognize and respond to physical cues during decision-making processes.
- This integration supported a more holistic approach to leadership, where physical sensations informed cognitive processes.

4. Enhanced Reflectivity:

- Participants engaged in reflective practices that allowed them to critically evaluate their habitual responses and develop new ways of thinking and acting.
- This reflectivity was crucial for personal and professional growth, enabling leaders to adapt to complex and uncertain environments.

5. Utilization of Creative Processes:

- The use of arts-based methods, such as working with clay, fostered creativity and imaginative thinking.
- Participants found that engaging in creative processes helped them access alternative perspectives and solutions.

Effects of Art-Based Approach:

- Development of Negative Capability: Ability to remain present and open in uncertainty.
- Increased Self-Awareness and Emotional Regulation: Enhanced recognition and management of emotional and physical responses.
- Integration of Body and Mind: Connection between physical sensations and cognitive processes.
- Enhanced Reflectivity: Critical evaluation of habitual responses and development of new approaches.
- Utilization of Creative Processes: Engagement in creative activities to access alternative perspectives.

Keywords for Data Summary Table

- Development of Negative Capability
- Increased self-awareness
- Emotional regulation
- Integration of body and mind
- Enhanced reflectivity
- Utilization of creative processes

These keywords capture the main outcomes of the study, highlighting the positive impacts of an art-based approach on leadership development, self-awareness, and emotional regulation.

Hurdle & Greenhaw (2023) [173]

Summary of Empirical Results and Effects of Art-Based Pedagogy from Hurdle & Greenhaw (2023) [173]

Empirical Results:

1. High Satisfaction with Assignment:

- Learners reported high levels of enjoyment with the film analysis assignment, indicating a mean score of 5.7 out of 7.
- The task was perceived as easy to complete, with a mean score of 6.0 for applying their understanding of group development stages.

2. Effective Learning Tool:

- Participants successfully identified and described the stages of group development (forming, storming, norming, performing, and adjourning) through the film "Jumanji: Welcome to the Jungle".
- The analysis showed that students could apply theoretical knowledge to practical scenarios depicted in the film.

3. Vicarious Learning:

- The film provided a vicarious learning experience, enabling students to observe and analyze leadership and team dynamics in a controlled, relatable context.
- Learners indicated that film helped them better understand leadership concepts compared to traditional methods.

Effects of Art-Based Approach:

- High Satisfaction with Assignment: Learners enjoyed the assignment and found it easy to apply theoretical concepts.
- Effective Learning Tool: Successful identification and understanding of group development stages.
- Vicarious Learning: Enhanced understanding of leadership concepts through observation and analysis of film scenarios.

Keywords for Data Summary Table

- High satisfaction with assignment
- Effective learning tool
- Vicarious learning

These keywords capture the main outcomes of the study, highlighting the positive impacts of using film as an art-based approach to teaching leadership and group development concepts.

Kaimal et al. (2014) [174]

Summary of Empirical Results and Effects of Art-Based Pedagogy from Kaimal et al. (2014) [174]

Empirical Results:

1. Enhanced Reflective Practice:

- Participants reported a deeper level of reflection on their leadership practices and personal development.
- The engagement with the arts encouraged leaders to pause, reflect, and gain new insights into their behaviors and decisions.

2. Increased Creativity and Innovation:

- The arts-based activities stimulated creative thinking and problem-solving skills.
- Participants felt more empowered to introduce innovative practices within their schools.

3. Empowerment and Agency:

- The experience of engaging in new and challenging activities empowered participants to take risks and explore new ways of leading.
- Participants gained a sense of agency, feeling more confident in their abilities to influence and drive change.

4. Broadened Perspectives on Leadership:

- The integration of arts-based pedagogy provided participants with a broader perspective on leadership, emphasizing the value of creativity, empathy, and adaptability.
- Leaders developed a more nuanced understanding of their roles and the impact of their leadership on the school community.

Effects of Art-Based Approach:

- Enhanced Reflective Practice: Increased depth of reflection on leadership and personal development.
- Increased Creativity and Innovation: Stimulated creative thinking and problem-solving skills.
- Empowerment and Agency: Encouraged risk-taking and exploration of new leadership approaches.
- Broadened Perspectives on Leadership: Provided a holistic view of leadership, emphasizing creativity, empathy, and adaptability.

Keywords for Data Summary Table

- Enhanced reflective practice
- Increased creativity
- Increased innovation
- Empowerment
- Increased agency
- Broadened perspectives on leadership

These keywords capture the main outcomes of the study, reflecting the positive impacts of an art-based approach on leadership development and effectiveness.

Kaimal et al. (2016) [175]

Summary of Empirical Results and Effects of Art-Based Pedagogy from Kaimal et al. (2016) [175]

Empirical Results:

1. Enhanced Reflective Practice:

- Participants reported significant improvements in their reflective practices. The art museum visits prompted leaders to consider multiple perspectives and engage in deep reflection about their leadership styles and decision-making processes.
- This reflective practice helped them understand the importance of seeing situations from different angles and considering diverse viewpoints in their leadership roles.

2. Increased Empathy and Understanding:

- The guided art museum sessions fostered greater empathy among participants. They developed a better understanding of the emotional and subjective experiences of others, which translated into more empathetic leadership practices.
- Participants reported that this empathy extended to their interactions with staff and students, improving their ability to connect and communicate effectively.

3. Broadened Perspectives on Leadership:

- The art-based activities broadened participants' perspectives on leadership. They learned to appreciate the value of creativity, flexibility, and openness in leadership.
- The experience encouraged leaders to integrate artistic and creative approaches into their leadership practices, promoting a more holistic and inclusive leadership style.

4. Empowerment:

- Participants felt empowered by the creative and reflective processes. The art-based approach boosted their confidence in their leadership abilities and encouraged them to take risks and innovate in their roles.
- This sense of empowerment translated into more proactive and dynamic leadership behaviors.

Effects of Art-Based Approach:

- Enhanced Reflective Practice: Improved ability to reflect on leadership styles and decision-making processes.
- Increased Empathy and Understanding: Greater empathy and better understanding of others' emotional experiences.
- Broadened Perspectives on Leadership: Appreciation of creativity, flexibility, and openness in leadership.
- Empowerment: Increased confidence and willingness to take risks and innovate.

Keywords for Data Summary Table

- Enhanced reflective practice
- Increased empathy
- Broadened perspectives on leadership
- Empowerment

These keywords capture the main outcomes of the study, reflecting the positive impacts of an art-based approach on leadership development and effectiveness.

“Some participants were enthused by their participation in these sessions and took it to their own schools and professional contexts. They translated the conceptual lessons from the session in order to create new ways to bring about change in their schools.”

- Transfer to professional context

Katz-Buonincontro (2011) [50]

Summary of Empirical Results and Effects of Art-Based Pedagogy from Katz-Buonincontro (2011) [50]

Empirical Results:

1. Emotional Catharsis:

Participants experienced a release of emotions, referred to as catharsis, through the role-playing process in improvisational theatre.

This emotional release was crucial in helping them confront and process workplace conflicts.

2. Enhanced Empathy:

The theatre exercises significantly enhanced participants' empathy towards others.

By embodying different roles, participants developed a better understanding of and sensitivity to the emotions and perspectives of others.

3. Heightened Sensory Perception:

Participants reported a heightened sense of sensory perception, which allowed them to be more attuned to their surroundings and the nuances of interpersonal interactions.

This heightened awareness contributed to a deeper engagement with the material and the experiences of others.

4. Reflective Thinking:

The art-based approach promoted deep reflective thinking among participants.

Participants critically reflected on their own behaviors, decisions, and leadership styles during and after the theatre exercises.

5. Increased Creativity and Problem-Solving:

The improvisational theatre activities fostered creativity and innovative thinking.

Participants learned to approach problems with a creative mindset, considering multiple perspectives and solutions.

6. Development of a Sense of Community:

The collaborative nature of the theatre exercises fostered a strong sense of community among participants.

They felt more connected to their peers and more effective in collaborative settings.

Effects of Art-Based Approach:

- Emotional Catharsis: The role-playing led to significant emotional release and helped participants process workplace conflicts.
- Enhanced Empathy: Participants developed a heightened sense of empathy towards others.
- Heightened Sensory Perception: Participants reported increased sensory awareness, enhancing their engagement and interaction skills.
- Reflective Thinking: The approach promoted deep reflective thinking, allowing for critical self-analysis and growth.
- Increased Creativity: The art-based pedagogy fostered creativity and innovative problem-solving skills.
- Sense of Community: The activities strengthened the sense of community and collaboration among participants.

Keywords for Data Summary Table

- Emotional catharsis
- Enhanced empathy
- Heightened sensory perception
- Reflective thinking
- Increased creativity
- Sense of community

These keywords reflect the main outcomes of the study, highlighting the positive impacts of an art-based approach to leadership development.

Katz-Buonincontro & Phillips (2011) [176]

Summary of Empirical Results and Effects of Art-Based Pedagogy from Katz-Buonincontro & Phillips (2011) [176]

Empirical Results:

1. Enhanced Reflectivity and Problem Framing:

Participants demonstrated increased reflective thinking and problem framing abilities. They were able to reframe problems and consider new, previously unrecognized elements and resources.

Reflection and journaling allowed participants to ponder problematic situations deeply and consider new possible courses of action.

2. Improved Problem-Solving Skills:

The improvisational theatre exercises promoted fast and interactive problem-solving. Participants quickly derived descriptions of workplace problems and explored multiple solutions.

The arts-based approach enabled participants to address problems in a creative and flexible manner, often generating multiple potential solutions.

3. Increased Creativity:

Emotional blocks or barriers that hindered problem-solving were addressed through arts-based activities, enabling clearer evaluation of problems.

Participants engaged in creative risk-taking and rediscovered their creative roots. This creativity was applied to problem-solving and leadership challenges.

The arts-based approach fostered a sense of creativity and innovation, helping participants to think outside the box.

4. Enhanced Empathy and Understanding:

Participants developed greater empathy and understanding towards others through role-playing and other artistic activities.

The process of creating and reflecting on art allowed them to better understand and relate to the experiences and perspectives of others.

Effects of Art-Based Approach:

- Enhanced Reflectivity: Participants developed a deeper ability to reflect on their actions and decisions, leading to better problem framing.
- Improved Problem-Solving: The approach promoted fast and flexible problem-solving skills, allowing for the generation of multiple solutions.
- Increased Creativity: The arts-based activities fostered creativity and innovation in addressing leadership challenges.

Keywords for Data Summary Table

- Enhanced reflectivity
- Improved problem-solving skills
- Increased creativity

These keywords reflect the main outcomes of the study, highlighting the positive impacts of an art-based approach to leadership development.

“Three interesting thematic patterns emerged: thoughtful reflection, increased risk-taking and a return to creative roots.”

- Increased risk-taking

“Nearly all talked about a heightened sense of visual perception.”

- Heightened sense of visual perception

Katz-Buonincontro et al. (2015) [177]

Summary of Empirical Results and Effects of Art-Based Pedagogy from Katz-Buonincontro et al. (2015) [177]

Empirical Results:

1. In-Depth, Authentic Personal Reflection:

- Participants engaged in deep, authentic personal reflection on their professional identity and leadership practices.
- The art-based activities, such as photo-captioning and art-making, prompted leaders to reflect on their roles, values, and behaviors in a meaningful way.

2. Amplified Attention to Everyday and Environmental Details:

- The study found that participants developed a heightened awareness of everyday and environmental details.
- This increased attention helped leaders become more observant and mindful in their professional contexts.

3. Increased Willingness to Take Risks:

- The creative and supportive environment encouraged participants to take more risks in their leadership practices.
- Leaders felt more confident to experiment with new ideas and approaches, fostering innovation and growth.

4. Return to Creative Roots:

- Engaging in artistic activities allowed participants to reconnect with their creative instincts and skills.
- This return to creative roots provided a fresh perspective on problem-solving and leadership.

5. Defining and Reflecting on Leadership Paradigms:

- The arts-based approach helped students define and reflect upon their own paradigms of leadership.
- Participants were able to articulate and examine their personal leadership philosophies and approaches.

6. Rich Understanding of Work Situations:

- The activities allowed students to generate a rich understanding of their work situations.
- Through artistic expression and discussion, participants gained deeper insights into their professional environments and challenges.

7. Making Inferences About Leadership Situations:

- The creative exercises offered students ways to make inferences about leadership situations and the purpose of their leadership practice.
- Participants were able to draw connections between their experiences and broader leadership concepts.

Effects of Art-Based Approach:

- In-Depth Personal Reflection: Facilitated deep reflection on professional identity and leadership practices.
- Increased Observational Skills: Amplified attention to everyday and environmental details.
- Encouraged Risk-Taking: Fostered a willingness to take risks and experiment with new ideas.
- Reconnection with Creativity: Enabled a return to creative roots and fresh perspectives on problem-solving.
- Leadership Paradigm Definition: Helped define and reflect on personal leadership paradigms.
- Enhanced Understanding: Provided a rich understanding of work situations.
- Insight into Leadership Practice: Enabled making inferences about leadership situations and practices.

Keywords for Data Summary Table

- In-depth personal reflection
- Increased observational skills
- Encouraged risk-taking
- Reconnection with creativity
- Leadership paradigm definition
- Enhanced understanding
- Insight into leadership practice

These keywords capture the main outcomes of the study, highlighting the positive impacts of an art-based approach on leadership development and effectiveness.

Kilic (2023) [40]

Summary of Empirical Results and Effects of Art-Based Pedagogy from Kilic (2023) [40]

Empirical Results:

1. Enhanced Creativity:

Participants showed significant improvement in their creativity, particularly in handling paradoxical and complex situations.

The arts-based activities encouraged both/and thinking, enabling leaders to integrate contradictory elements and generate innovative solutions.

2. Improved Communication Skills:

There was a notable improvement in both verbal and non-verbal communication skills.

Emotional (non-verbal) communication skills increased more significantly than social (verbal) skills.

3. Healing Effect:

Participants reported a substantial positive change in their mood and energy levels due to the arts involvement.

The affect grid scores indicated a 25% increase in feelings and a 29% increase in arousal, reflecting a heightened sense of well-being and reduced stress.

4. Increased Social Sensitivity:

Participants developed a greater ability to perceive and understand the feelings and viewpoints of others.

The social sensitivity scores showed an overall increase, with participants becoming more empathetic and inclusive.

5. Enhanced Reflectivity and Self-Awareness:

Participants engaged deeply in reflective thinking, critically evaluating their behaviors and leadership styles.

The process facilitated increased self-awareness and personal growth.

6. Strengthened Sense of Community:

The collaborative nature of the arts-based activities fostered a strong sense of community and teamwork among participants.

Participants felt more connected to their peers and more effective in collaborative settings.

Effects of Art-Based Approach:

- Enhanced Creativity: Improved ability to think creatively and handle paradoxical situations.
- Improved Communication Skills: Increased proficiency in both verbal and non-verbal communication.
- Healing Effect: Positive changes in mood and energy levels, contributing to overall well-being.
- Increased Social Sensitivity: Greater empathy and understanding towards others.
- Enhanced Reflectivity: Improved self-awareness and reflective thinking.
- Sense of Community: Stronger sense of teamwork and collaboration.

Keywords for Data Summary Table

- Enhanced creativity
- Improved communication skills
- Healing effect
- Increased social sensitivity
- Enhanced reflectivity
- Sense of community

These keywords reflect the main outcomes of the study, highlighting the positive impacts of an art-based approach to leadership development.

Leonard et al. (2013) [106]

Summary of Empirical Results and Effects of Art-Based Pedagogy from Leonard et al. (2013) [106]

Empirical Results:

1. Enhanced Reflective Learning:

- Participants engaged in reflective learning, examining their leadership styles and professional roles through the experience of playing Gamelan music.

- The activity provided a new perspective on their behaviors and decisions, promoting critical self-evaluation.

2. Increased Emotional Engagement:

- The Gamelan sessions elicited a range of emotional responses, from anxiety to tranquility, which helped participants become more aware of their emotional states.

- This emotional engagement was seen as beneficial for developing self-awareness and empathy.

3. Improved Teamwork and Collaboration:

- Learning and performing Gamelan music fostered strong teamwork and collaboration among participants.

- Participants recognized the importance of relying on each other, building trust, and working together harmoniously.

4. Increased Willingness to Take Risks:

- Participants reported a greater willingness to take risks and step out of their comfort zones during the sessions.

- The safe and supportive environment encouraged experimentation and innovation in their practices.

Effects of Art-Based Approach:

- Enhanced Reflective Learning: Promoted critical self-evaluation and reflection on leadership styles.

- Increased Emotional Engagement: Heightened awareness of emotional states and development of empathy.

- Improved Teamwork and Collaboration: Fostered strong teamwork and collaborative skills.

- Promotion of Equality and Co-Production: Emphasized democratic participation and equality.

- Increased Willingness to Take Risks: Encouraged experimentation and innovation.

Keywords for Data Summary Table

- Enhanced reflective learning
- Increased emotional engagement
- Improved teamwork
- Improved collaboration
- Increased willingness to take risks

These keywords reflect the main outcomes of the study, highlighting the positive impacts of an art-based approach on leadership development and effectiveness.

"The activity generated ideas for transferring the learning from the session to the 'real world'."

- Learning transfer to real world

Medeiros et al. (2012) [178]

Summary of Empirical Results and Effects of Art-Based Pedagogy from Medeiros et al. (2012) [178]

Empirical Results:

1. Development of Ethical Humanist Skills and Professionalism:

- The "Imitating Art" workshop significantly expanded students' views on ethical humanist attitudes, helping them to internalize and reflect on these values through the creative process.
- Two-thirds of the students indicated that the workshop supported the development of essential competencies outlined in the Brazilian national curriculum guidelines (NCG), such as health care, decision-making, communication, leadership, administration, and teamwork.

2. Enhanced Teamwork, Leadership, and Communication Skills:

- 90% of students specifically noted improvements in teamwork, leadership, decision-making, communication, and professionalism as a result of the workshop.
- The hands-on, creative activities involved in reproducing and reflecting on art masterpieces helped students to better understand and practice these crucial skills.

Effects of Art-Based Approach:

- Enhanced Ethical Humanist Skills: Expanded views on ethical humanist attitudes and professionalism.
- Improved Teamwork and Leadership: Significant development in teamwork, leadership, and communication skills.

Keywords for Data Summary Table

- Enhanced ethical humanist skills
- Improved teamwork
- Improved leadership
- Improved communication

These keywords capture the main outcomes of the study, highlighting the positive impacts of an art-based approach on medical education and the development of essential professional competencies.

Munro et al. (2015) [179]

Summary of Empirical Results and Effects of Art-Based Pedagogy from Munro et al. (2015) [179]

Empirical Results:

1. Heightened Awareness of Emotions and Feelings:

- Participants developed a greater awareness of the role of emotions and feelings in business communication.
- This increased emotional intelligence contributed to more effective interpersonal interactions and leadership.

2. Awareness of Cognitive and Sensory Preferences:

- The study highlighted that participants became more aware of their cognitive and sensory preferences.
- Understanding these preferences helped participants tailor their communication strategies to be more effective.

3. Enhanced Communication Effectiveness:

- As a result of the heightened awareness of emotional, cognitive, and sensory preferences, participants experienced enhanced communication effectiveness.
- This improvement was reflected in their ability to convey ideas and emotions more clearly and effectively.

4. Recognition of Habitual Communication Modes:

- Participants identified their habitually preferred modes of communication and learned to adapt these modes to different contexts.
- This flexibility contributed to more dynamic and adaptive communication strategies.

Effects of Art-Based Approach:

- Heightened Emotional Awareness: Greater understanding of the role of emotions in communication.
- Increased Awareness of Preferences: Recognition of cognitive and sensory preferences.
- Enhanced Communication Effectiveness: Improved clarity and effectiveness in communication.
- Adaptability in Communication Modes: Flexibility in using different communication modes based on context.

Keywords for Data Summary Table

- Heightened emotional awareness
- Increased awareness of cognitive and sensory preferences
- Enhanced communication effectiveness
- Adaptability in communication modes

These keywords reflect the main outcomes of the study, highlighting the positive impacts of an art-based approach on communication skills and leadership development.

“The overall outcome indicated an increase in emotional competency in the group.”

- Increased emotional competency

Parush & Koivunen (2014) [68]

Summary of Empirical Results and Effects of Art-Based Pedagogy from Parush & Koivunen (2014) [68]

Empirical Results:

1. Heightened Aesthetic Pleasure and Memorability:

- Participants derived significant aesthetic pleasure from the workshops, with many describing the experience as memorable and powerful.
- Feedback highlighted the lasting impact of the sessions, which remained vivid in participants' memories.

2. Increased Self-Confidence and Risk-Taking:

- Many participants noted an increase in self-confidence as they stepped out of their comfort zones to conduct a choir.
- The experience encouraged leaders to take risks and embrace new challenges, resulting in personal growth and a sense of achievement.

Effects of Art-Based Approach:

- Heightened Aesthetic Pleasure: Significant enjoyment and memorability from the aesthetic aspects of the workshops.
- Increased Self-Confidence and Risk-Taking: Greater confidence and willingness to take risks.

Keywords for Data Summary Table

- Heightened aesthetic pleasure
- Memorability
- Increased self-confidence
- Increased risk-taking

These keywords reflect the main outcomes of the study, highlighting the positive impacts of an art-based approach on leadership development and communication effectiveness.

Peña & Grant (2019) [180]

Summary of Empirical Results and Effects of Art-Based Pedagogy from Peña & Grant (2019) [180]

Empirical Results:

1. Disorienting Dilemma:

The art exercise presented a disorienting dilemma that challenged students' existing beliefs and assumptions.

Students reported that the experience made them question their preconceived notions and led to significant changes in their perspectives.

2. Sense-Making:

The art-based activity helped students make sense of complex and ambiguous situations.

Participants had "ah-ha" moments where they realized new possibilities and gained insights into their own creativity and problem-solving abilities.

3. Self-Awareness:

The exercise significantly increased participants' self-awareness.

Students reflected on their strengths, weaknesses, thoughts, and emotions, leading to a better understanding of themselves and their leadership capabilities.

4. Self-Efficacy:

The activity enhanced students' belief in their ability to produce creative outcomes (creative self-efficacy).

Participants felt more confident in their creative abilities and were motivated to engage in creative activities.

Effects of Art-Based Approach:

- Enhanced Reflectivity: Participants developed greater reflective thinking skills, allowing for critical self-analysis and personal growth.
- Improved Empathy: The approach fostered empathy and understanding towards others.
- Enhanced Creativity: The art-based pedagogy encouraged creative risk-taking and innovative thinking.
- Increased Self-Efficacy: Participants felt more confident in their creative abilities and leadership skills.
- Greater Self-Awareness: The exercise helped students understand their own emotions, thoughts, and leadership styles better.

Keywords for Data Summary Table

- Disorienting dilemma
- Sense-making
- Self-awareness
- Self-efficacy

These keywords reflect the main outcomes of the study, highlighting the positive impacts of an art-based approach to leadership development.

Rajendran & Andrew (2014) [51]

Summary of Empirical Results and Effects of Art-Based Pedagogy from Rajendran & Andrew (2014) [51]

Empirical Results:

1. Enhanced Memorization and Understanding:

- The visual dimension of films catered to students with different learning styles, leading to easier memorization of leadership concepts.
- Students found it easier to reconceptualize theories presented in films compared to traditional lecture notes.

2. Contextual Understanding and Application:

- Films provided quasi-authentic contexts where leadership facets could be observed and understood in action.
- Students were able to see leadership theories depicted in real-life scenarios, aiding in the practical application of these concepts.

3. Learner Autonomy:

- The use of film encouraged learner autonomy, with students independently applying theoretical concepts to film scenarios.
- This method promoted self-directed learning and enhanced critical thinking skills.

4. Engagement and Motivation:

- The use of films made learning more engaging and enjoyable, which motivated students to invest more effort in their studies.
- Students appreciated the dynamic and interactive nature of learning through films.

5. Practical Learning Experience:

- Students reported that films provided a richer, more attractive learning experience compared to traditional methods.
- The practical application of leadership models through film helped bridge the gap between theory and real-world practice.

Effects of Art-Based Approach:

- Enhanced Memorization and Understanding: Easier memorization and reconceptualization of leadership concepts.
- Contextual Understanding: Practical understanding of leadership theories in real-life contexts.
- Learner Autonomy: Promoted self-directed learning and critical thinking.
- Engagement and Motivation: Increased motivation and engagement in learning.
- Practical Learning Experience: Richer, more attractive learning experience with practical applications.

Keywords for Data Summary Table

- Enhanced memorization
- Improved understanding
- Contextual understanding
- Learner autonomy
- Engagement and motivation
- Practical learning experience

These keywords reflect the main outcomes of the study, highlighting the positive impacts of using films as an art-based approach to teaching leadership effectiveness.

Romanowska et al. (2011) [67]

Summary of Empirical Results and Effects of Art-Based Pedagogy from Romanowska et al. (2011) [67]

Empirical Results:

1. Improved Mental Health:

The art-based leadership program (Schibbolet group) showed significant improvements in mental health compared to the conventional group.

There was a significant decrease in total poor mental health scores, which included measures of emotional exhaustion, sleep disturbances, and depressive symptoms.

2. Enhanced Coping Strategies:

Participants in the Schibbolet group demonstrated improved coping mechanisms, with a significant reduction in covert coping behaviors.

Improved coping strategies were linked to better handling of stress and work-related challenges.

3. Better Performance-Based Self-Esteem:

The art-based approach led to a significant improvement in performance-based self-esteem.

Participants felt more confident in their abilities and less dependent on external validation for their self-worth.

4. Favorable Biological Outcomes:

There was a significant interaction effect for serum DHEA-s, with the Schibbolet group showing a smaller decrease in DHEA-s concentration compared to the conventional group.

This suggests a protective effect of the art-based program against stress-related biological deterioration.

5. Reduction in Stress Indicators:

The Schibbolet group exhibited lower levels of stress-related symptoms and better overall well-being.

The improvements in both mental and biological indicators suggest a comprehensive positive impact on stress management.

Effects of Art-Based Approach:

- Improved Mental Health: Significant improvements in emotional exhaustion, sleep disturbances, and depressive symptoms.

- Enhanced Coping Strategies: Reduction in covert coping and improved handling of stress.

- Better Performance-Based Self-Esteem: Increased confidence and reduced dependence on external validation.

- Favorable Biological Outcomes: Protection against stress-related biological deterioration, evidenced by stable DHEA-s levels.

- Reduction in Stress Indicators: Overall reduction in stress and better well-being.

Keywords for Data Summary Table

- Improved mental health
- Enhanced coping strategies
- Better performance-based self-esteem
- Favorable biological outcomes
- Reduction in stress indicators

These keywords reflect the main outcomes of the study, highlighting the positive impacts of an art-based approach to leadership development.

Romanowska et al. (2013) [32]

Summary of Empirical Results and Effects of Art-Based Pedagogy from Romanowska et al. (2013) [32]

Empirical Results:

1. Improvement in Sense of Coherence (SOC):

The art-based leadership program (Shibboleth group) led to a significant increase in participants' Sense of Coherence, which is a measure of how comprehensible, manageable, and meaningful they find their lives.

Participants in the Shibboleth group showed increased psychological resilience to stress compared to those in the conventional program.

2. Increase in Agreeableness:

Participants in the art-based group demonstrated a significant increase in Agreeableness, a personality trait associated with being cooperative, compassionate, and understanding towards others.

This improvement suggests enhanced pro-social behavior among the participants.

3. Reduction in Laissez-Faire Leadership:

There was a notable decrease in Laissez-faire leadership behavior in the Shibboleth group. Laissez-faire leadership is characterized by a lack of proactive leadership and responsibility.

Participants became more engaged and took on more leadership responsibilities.

4. Enhanced Capacity to Cope with Stress:

The Shibboleth group showed a significant improvement in their ability to cope with stress. This included both problem-focused and emotion-focused coping strategies.

Participants were better equipped to handle stress and manage challenging situations effectively.

Effects of Art-Based Approach:

- Improved Mental Resilience: Increased Sense of Coherence indicates better mental resilience and a more positive outlook on life.

- Enhanced Pro-Social Behavior: Increased Agreeableness reflects better social interactions and empathy towards others.

- Reduced Passive Leadership: Decrease in Laissez-faire behavior shows a shift towards more active and responsible leadership.

- Better Stress Management: Improved Capacity to Cope with stress highlights enhanced skills in managing both personal and professional stressors.

Keywords for Data Summary Table

- Improved mental resilience
- Enhanced pro-social behavior
- Reduced passive leadership
- Better stress management

These keywords reflect the main outcomes of the study, highlighting the positive impacts of an art-based approach to leadership development.

Romanowska et al. (2014) [79]

Summary of Empirical Results and Effects of Art-Based Pedagogy from Romanowska et al. (2014) [79]

Empirical Results:

1. Improved Self-Awareness and Perceptual Alignment:

The art-based leadership intervention significantly improved leaders' self-awareness and humility.

Leaders in the art-based group showed increased alignment between their self-perceptions and subordinates' perceptions, indicating a reduction in self-overrating and hubris.

2. Reduction in Laissez-Faire Leadership:

Participants in the art-based group exhibited a significant reduction in laissez-faire leadership behaviors.

This indicates an increase in active engagement, decision-making, and responsibility-taking by the leaders.

3. Enhanced Capacity to Cope with Stress:

Leaders in the art-based group demonstrated an improved capacity to cope with stress.

This was measured by both self-ratings and subordinate ratings, showing a significant improvement compared to the conventional leadership training group.

4. Positive Impact on Subordinates:

Subordinates of leaders in the art-based group reported better overall well-being and job satisfaction.

There were significant improvements in subordinates' perceptions of their leaders' effectiveness and supportiveness.

5. Improved Leader Performance:

Overall, leaders in the art-based intervention group showed improved performance in their leadership roles.

This was evidenced by higher ratings from subordinates and better self-assessments post-intervention.

Effects of Art-Based Approach:

- Improved Self-Awareness and Perceptual Alignment: Significant improvement in leaders' self-awareness and alignment with subordinates' perceptions.
- Reduction in Passive Leadership: Decrease in laissez-faire behaviors, indicating more proactive and responsible leadership.
- Better Stress Management: Enhanced capacity to cope with stress and manage challenging situations.
- Positive Impact on Subordinates: Improved well-being and job satisfaction among subordinates.
- Enhanced Leader Performance: Overall improvement in leadership performance as rated by both leaders and subordinates.

Keywords for Data Summary Table

- Improved self-awareness
- Enhanced perceptual alignment
- Reduction in passive leadership
- Better stress management
- Positive impact on subordinates
- Improved leader performance

These keywords reflect the main outcomes of the study, highlighting the positive impacts of an art-based approach to leadership development.

Sandberg et al. (2023) [181]

Summary of Empirical Results and Effects of Art-Based Pedagogy from Sandberg et al. (2023) [181]

Empirical Results:

1. Improved Physical Presence:

Participants showed significant and prolonged improvement in physical presence, which was noticeable and persisted in their everyday working lives.

This was evidenced by both self-assessment and external observations.

2. Enhanced Nonverbal Communication:

The art-based approach sensitized participants to nonverbal communication, shaping mutual interactions positively.

Participants became more aware of their posture, movement, and bodily expressions, which improved their overall communication effectiveness.

3. Positive Aesthetic Experience:

The aesthetic experience of the workshop had an inherent learning effect, which was further enhanced through reflection and repetition of exercises.

Participants found the joint aesthetic experience to be a significant factor in their learning process.

4. Increased Sensitivity and Attention:

Participants reported increased sensitivity to their own and others' physical presence and body language.

This heightened awareness translated into better attention to others in professional settings.

5. Transfer of Learning to Professional Practice:

Participants were able to transfer the skills learned in the workshop to their professional contexts.

The study found a positive correlation between the design of the art-based workshop and the successful transfer of skills to the workplace.

Effects of Art-Based Approach:

- Improved Physical Presence: Enhanced ability to present oneself effectively in professional settings.
- Enhanced Nonverbal Communication: Better awareness and use of nonverbal cues in communication.
- Positive Aesthetic Experience: Aesthetic elements of the workshop contributed significantly to learning.
- Increased Sensitivity and Attention: Greater sensitivity to physical and nonverbal aspects of interactions.
- Successful Learning Transfer: Effective application of learned skills in professional practice.

Keywords for Data Summary Table

- Improved physical presence
- Enhanced nonverbal communication
- Positive aesthetic experience
- Increased sensitivity
- Successful learning transfer

These keywords reflect the main outcomes of the study, highlighting the positive impacts of an art-based approach to leadership development.

Schyns et al. (2013) [35]

Summary of Empirical Results and Effects of Art-Based Pedagogy from Schyns et al. (2013) [35]

Empirical Results:

1. Heightened Self-Reflection:

- Participants engaged in deep self-reflection through drawing exercises.
- This reflective practice facilitated a better understanding of their implicit leadership theories.

2. Enhanced Self-Awareness:

- The drawing exercises heightened participants' self-awareness, allowing them to recognize their own implicit beliefs about leadership.
- Participants gained insights into their own strengths and weaknesses as leaders.

Effects of Art-Based Approach:

- Heightened Self-Reflection: Facilitated a better understanding of implicit leadership theories.
- Enhanced Self-Awareness: Increased recognition of implicit beliefs about leadership.

Keywords for Data Summary Table

- Heightened self-reflection
- Enhanced self-awareness

These keywords reflect the main outcomes of the study, highlighting the positive impacts of an art-based approach on leadership development and self-awareness.

Singh & Widén (2020) [182]

Summary of Empirical Results and Effects of Art-Based Pedagogy from Singh & Widén (2020) [182]

Empirical Results:

1. Leadership Perspective Changes:

The study revealed that 87% of students experienced a change or reinforcement in their perspectives on leadership after engaging with the film-based pedagogy.

60.4% of students reported an evolved perspective on leadership, while 26.7% found their existing beliefs reinforced.

Only 12.9% of students felt their leadership perspectives remained unchanged.

2. Critical Thinking and Analytical Skills:

The activity significantly enhanced students' critical thinking skills, enabling them to analyze various leadership styles and behaviors depicted in the movies.

Students demonstrated the ability to connect theoretical leadership concepts with practical examples from the films.

3. Engagement with Leadership Concepts:

The film-based approach provided students with a more engaging and realistic understanding of leadership concepts.

Students were able to identify various leadership styles such as transformational, authoritative, democratic, and collaborative leadership.

They analyzed the strengths and weaknesses of these styles as portrayed by characters in the movies.

4. Enhanced Understanding of Leadership Traits:

The exercise helped students recognize important leadership traits such as empathy, resilience, communication, and ethical behavior.

Students reported a greater appreciation for the complexity of leadership and the importance of context in leadership effectiveness.

5. Application of Learning:

Many students indicated that they could realistically apply the leadership lessons learned from the movies to their current and future professional situations.

The film-based pedagogy encouraged students to reflect on their own leadership styles and consider areas for personal improvement.

Effects of Art-Based Approach:

- Changed Leadership Perspectives: Significant shift or reinforcement in students' leadership perspectives.
- Improved Critical Thinking: Enhanced ability to critically analyze leadership behaviors and styles.
- Engaged Learning: Increased engagement with leadership concepts through an immersive and realistic medium.
- Recognition of Key Traits: Better understanding of essential leadership traits and their application.
- Practical Application: Ability to apply learned concepts to real-world professional settings.

Keywords for Data Summary Table

- Changed leadership perspectives
- Improved critical thinking
- Engaged learning
- Recognition of essential leadership traits
- Practical application

These keywords reflect the main outcomes of the study, highlighting the positive impacts of an art-based approach to leadership development.

Sutherland (2012) [183]

Summary of Empirical Results from Sutherland (2012) [183]

Empirical Results

1. Enhanced Reflexivity: Participants developed deeper reflexive capabilities, enabling them to critically evaluate their leadership practices and personal development.

2. Aesthetic Engagement: The art-based approach fostered a high level of aesthetic engagement, making learning experiences more memorable and impactful.

3. Increased Emotional Awareness: Participants reported a heightened awareness of their emotions and those of others, improving their emotional intelligence in leadership contexts.

4. Improved Self-Awareness: There was significant growth in participants' self-awareness, helping them understand their strengths and weaknesses in leadership roles.

5. Memorable Learning: The unique, non-routine learning environment created lasting memories that participants could draw upon in their future professional practices.

Keywords for Data Summary Table

- Enhanced reflexivity
- High aesthetic engagement
- Increased emotional awareness
- Improved self-awareness
- Memorable learning experiences

These results highlight the significant positive impact of art-based pedagogy on leadership development, emphasizing the importance of reflexivity, emotional intelligence, and memorable learning experiences in enhancing leadership capabilities.

“This continuing activity gets to the second emerging point, that of momentum – the ability of arts-based learning to influence future actions.”

“The arts-based experience has been transformed as an organising medium to structure professional context, ideas, discourse and actions.”

- Reconsidering future leadership practice

Sutherland & Jelinek (2015) [22]

Summary of Empirical Results and Effects of Art-Based Pedagogy from Sutherland & Jelinek (2015) [22]

Empirical Results

1. Relational Dynamics and Leadership

- Participants experienced a heightened awareness of the relational aspects of leadership. They felt strong connections with others, enhancing their understanding of leadership as an interactive and emotional process.

2. Power and Responsibility

- The workshops provided participants with a deeper understanding of the nature of power and responsibility in leadership. This was often a revelatory experience, making them reconsider their approach to leadership and control.

3. Long-term Impact on Professional Practice

- The workshops had a lasting impact on the professional practices of participants, with follow-up interviews showing that many had incorporated their new insights into their daily work, emphasizing listening, connecting, and supporting their teams over directive control.

Effects of the Art-Based Approach

- Emotional Engagement: The workshops created a significant emotional engagement, leading to enhanced motivation and inspiration among participants.

- Reflective Practice: Participants developed a more reflective approach to their leadership practices, often re-evaluating their actions and strategies based on the workshop experiences.

Keywords for Data Summary Table

- Heightened awareness of relational dynamics
- Deeper understanding of power and responsibility
- Long-term impact on professional practice
- Emotional engagement
- Reflective practice

These keywords capture the essence of the study's findings and provide a concise summary of the outcomes of the art-based pedagogy approach.

"However, although most found the entry of the choir to be highly significant, that trigger and consequent aesthetic experience was the beginning, an entry point into a new learning opportunity."

"The work of sensemaking began with attention grabbing sensory triggers that brought aesthetic experience into relief."

- Aesthetic experience
- Sense-making

"They developed deeper understandings of the relational nature of their professional practice, breaking the bonds of absolute boss-focused leadership."

"As learning outcomes, these relational and responsibility-oriented insights became part of a refinement of their professional practice, developing more people focused, humanistic qualities."

- Enhanced humanistic qualities

Winther (2018) [184]

Summary of Empirical Results and Effects of Art-Based Pedagogy from Winther (2018) [184]

Effects of the Art-Based Approach

Increased Self-confidence: Moving from lack of competence and resistance to mastering teaching despite initial self-experienced inadequacy.

Increased Sensitivity: Developing heightened sensitivity and awareness.

Improved Self-Contact: Improving connection with oneself.

Somatic Awareness: Enhancing awareness of bodily sensations and movements.

Keywords for Data Summary Table

- Increased self-confidence
- Increased sensitivity
- Improved self-contact
- Somatic awareness

These keywords reflect the main outcomes of the study, highlighting the positive impacts of an art-based approach on overcoming inadequacy, sensitivity, self-contact, and somatic awareness.

"The film shows many small joyful and intense situations in which they really succeed in their embodied professional leadership."

- Embodied leadership

Winther & Højlund Larsen (2022) [185]

Summary of Empirical Results and Effects of Art-Based Pedagogy from Winther & Højlund Larsen (2022) [185]

Empirical Results:

1. Increased Embodied Leadership Competence:

Participants developed a heightened awareness of their physical presence and how their bodies communicate in leadership contexts.

Leaders became more conscious of their somatic responses and how to use them to enhance leadership effectiveness.

2. Enhanced Emotional Awareness and Management:

The training facilitated a better understanding and management of emotions such as fear, sadness, anger, joy, and love.

Participants reported being able to harness these emotions constructively in their leadership practices.

3. Improved Reflexivity and Self-Contact:

Leaders gained tools to improve self-contact and somatic awareness, leading to a more grounded and present leadership style.

Reflective writing combined with dance activities provided deep insights into their leadership behaviors and personal growth.

4. Enhanced Communication Skills:

The study improved leaders' ability to read and respond to non-verbal cues, fostering better communication and relational dynamics.

Participants reported improved trust and openness in their interactions with team members.

5. Development of Relational Leadership Skills:

The dance exercises enhanced participants' ability to create and hold space, fostering a sense of presence and authority.

Leaders learned to balance leading and following, which enhanced collaborative efforts and team cohesion.

6. Sustained Personal and Professional Growth:

Participants noted that the benefits of the training extended beyond the course, contributing to ongoing personal and professional development.

The integration of dance and reflective writing created a sustainable model for continuous improvement in leadership practices.

Effects of Art-Based Approach:

- Increased Embodied Leadership Competence: Heightened awareness and effective use of physical presence.
- Enhanced Emotional Awareness: Better understanding and management of emotions in leadership.
- Improved Reflexivity: Deeper self-awareness and reflective practice.
- Enhanced Communication Skills: Improved ability to read and respond to non-verbal cues.
- Development of Relational Skills: Enhanced ability to create and hold space, balancing leading and following.
- Sustained Growth: Ongoing personal and professional development through embodied and reflective practices.

Keywords for Data Summary Table

- Increased embodied leadership competence
- Enhanced emotional awareness
- Improved reflexivity
- Enhanced communication skills
- Development of relational skills
- Sustained growth

These keywords reflect the main outcomes of the study, highlighting the positive impacts of an art-based approach to leadership development.

“Many leaders experienced important insights about … self-contact and somatic awareness.”

- Improved self-contact
- Somatic awareness

Woods et al. (2023) [45]

Summary of Empirical Results and Effects of Art-Based Pedagogy from Woods et al. (2023) [45]

Empirical Results:

1. Enhanced Aesthetic Qualities:

The study reported significant improvement in participants' aesthetic qualities, including aesthetic awareness and aesthetic reflexivity.

Participants developed a greater sensitivity to the aesthetic aspects of their leadership practice, leading to a richer and more nuanced understanding of their roles.

2. Improved Collaborative Leadership Capabilities:

Participants experienced growth in relational capabilities and communicative virtues, which are essential for effective distributed leadership.

The workshops facilitated a deeper understanding of the importance of relationships and communication in leadership, enhancing participants' ability to lead collaboratively.

3. Transformation of Professional Practice:

The art-based and embodied approaches led to transformative professional learning experiences.

Participants reported a shift in their professional practice, with a greater focus on integrating aesthetic and embodied learning into their leadership strategies.

4. Increased Reflexivity and Self-Awareness:

The workshops fostered a high degree of individual and collaborative reflexivity.

Participants became more self-aware and reflective about their leadership practices, leading to personal and professional growth.

Effects of Art-Based Approach:

- Enhanced Aesthetic Qualities: Improved aesthetic awareness and reflexivity, leading to a richer understanding of leadership.
- Improved Collaborative Leadership Capabilities: Growth in relational capabilities and communicative virtues.
- Transformative Learning: Shifts in professional practice and integration of aesthetic and embodied learning.
- Increased Reflexivity: Greater self-awareness and reflective practice.

Keywords for Data Summary Table

- Enhanced aesthetic awareness
- Improved collaborative leadership capabilities
- Transformative learning
- Increased reflexivity

These keywords reflect the main outcomes of the study, highlighting the positive impacts of an art-based approach to leadership development.

“Change was most evident in relation to: aesthetic qualities (aesthetic awareness, self-orientated awareness and other-orientated awareness ..., collaborative leadership capabilities (namely, relational capabilities and communicative virtues ..., view of leadership, which included widening perspectives on leadership and awareness of distributed leadership.”

- Self-awareness
- Interpersonal awareness
- Widening perspectives on leadership
- Increasing capacity for pro-active agency
