# Supplementary material for: Outcomes of Art-Based Leadership Development: A Qualitative Metasummary
Source: Behav Sci (Basel). 2024 Aug 14;14(8):714. doi: 10.3390/bs14080714 (PMC11351991; doi:10.3390/bs14080714)
Supplement: Supplementary file 1 [file behavsci-14-00714-s001.zip › Table S1_Search Strategy.pdf]

Table S1. Search strategy.

Last Run Via	Query	Limiters/Expanders	Results
Interface<br>- EBSCOhost Research Databases<br>Search Screen<br>- Advanced Search<br>Database<br>- Business Source Complete<br>Date<br>- 06.04.2024	S1<br>TI leadership AND TI (development OR training OR education OR pedagogy OR learning) AND TI (art OR arts OR painting OR sculpture OR music OR dance OR drama OR poetry OR movie)<br>S2<br>AB leadership AND AB (development OR training OR education OR pedagogy OR learning) AND AB (art OR arts OR painting OR sculpture OR music OR dance OR drama OR poetry OR movie)<br>S3<br>(S1 OR S2)	Limiters - Peer Reviewed; Publication Date: 20040101-20231231; Publication Type: Academic Journal; Document Type: Article; Language: English<br>Expanders - Apply equivalent subjects<br>Search modes - Boolean/Phrase	169
Interface<br>- EBSCOhost Research Databases<br>Search Screen<br>- Advanced Search<br>Database<br>- ERIC<br>Date<br>- 08.04.2024	S1<br>TI leadership AND TI (development OR training OR education OR pedagogy OR learning) AND TI (art OR arts OR painting OR sculpture OR music OR dance OR drama OR poetry OR movie)<br>S2<br>AB leadership AND AB (development OR training OR education OR pedagogy OR learning) AND AB (art OR arts OR painting OR sculpture OR music OR dance OR drama OR poetry OR movie)<br>S3<br>[S1] OR [S2]	Limiters Published Date: 20040101-20231231<br>Expanders Apply equivalent subjects<br>Search modes Boolean/Phrase	847
Interface<br>- EBSCOhost Research Databases<br>Search Screen<br>- Advanced Search<br>Database<br>- APA PsycInfo<br>Date<br>- 08.04.2024	S1<br>TI leadership AND TI (developmentOR training OR education ORpedagogy OR learning) AND TI (artOR arts OR painting OR sculpture ORmusic OR dance OR drama ORpoetry OR movie)<br>S2<br>AB leadership AND AB (developmentOR training OR education ORpedagogy OR learning) AND AB (artOR arts OR painting OR sculpture ORmusic OR dance OR drama ORpoetry OR movie)<br>S3<br>(S1 OR S2)	Limiters: Publication Date: 20040101-20231231; Publication Type: PeerReviewed Journal; Language: English; Document Type: JournalArticle<br>Expanders: Apply equivalent subjects<br>Search modes: Boolean/Phrase	224
Interface<br>- Hellenic Academic Libraries Link<br>Search Screen<br>- Advanced Search<br>Database<br>- Scopus<br>Date<br>- 06.04.2024	(TITLE(leadership) AND TITLE(development OR training OR education OR pedagogy OR learning) AND TITLE(art OR arts OR painting OR sculpture OR music OR dance OR drama OR poetry OR movie))<br>(ABS(leadership) AND ABS(development OR training OR education OR pedagogy OR learning) AND ABS(art OR arts OR painting OR sculpture OR music OR dance OR drama OR poetry OR movie))	PUBYEAR > 2003 AND PUBYEAR < 2024 AND (LIMIT-TO (DOCTYPE,"ar")) AND (LIMIT-TO (LANGUAGE,"English")) AND (LIMIT-TO (SRCTYPE,"j"))	795

Table S1. *Cont.*

Last Run Via	Query	Limiters/Expanders	Results
Interface<br>- Web of Science<br>Search Screen<br>- Advanced Search<br>Database<br>- Web of Science Core Collection<br>Date<br>- 08.04.2024	S1<br>leadership (Title) AND development OR training OR education OR pedagogy OR learning (Title) AND art OR arts OR painting OR sculpture OR music OR dance OR drama OR poetry OR movie (Title)<br>S2<br>leadership (Abstract) AND development OR training OR education OR pedagogy OR learning (Abstract) AND art OR arts OR painting OR sculpture OR music OR dance OR drama OR poetry OR movie (Abstract)<br>S3:<br>#2 OR #1	Article (Document Types) and English (Languages) Timespan: 2004-01-01 to 2023-12-31	525
Search engine<br>- Google Scholar<br>Date<br>- 14.04.2024	study AND "leadership development" AND arts<br>study AND "leadership development" AND painting<br>study AND "leadership development" AND sculpture<br>study AND "leadership development" AND music<br>study AND "leadership development" AND dance<br>study AND "leadership development" AND drama<br>study AND "leadership development" AND theatre<br>study AND "leadership development" AND poetry<br>study AND "leadership development" AND movie	Full text search<br>2004 – 2023<br>Sorted by relevance	169
