# Supplementary material for: Outcomes of Art-Based Leadership Development: A Qualitative Metasummary
Source: Behav Sci (Basel). 2024 Aug 14;14(8):714. doi: 10.3390/bs14080714 (PMC11351991; doi:10.3390/bs14080714)
Supplement: Supplementary file 1 [file behavsci-14-00714-s001.zip › Table S2_Thematic Hierarchy.pdf]

Table S2. Thematic hierarchy.

**Learner engagement and satisfaction (15)**		
Engagement (5)	Enhanced engagement (1)	Andenoro & Ward (2008) [166]
	Greater engagement (1)	Cranston & Kusanovich (2014) [168]
	Engagement and motivation (1)	Rajendran & Andrew (2014) [51]
	Emotional engagement (1)	Sutherland & Jelinek (2015) [22]
	Engaged learning (1)	Singh & Widén (2020) [182]
Aesthetic experience (5)	Appreciation for the arts (1)	Harz et al. (2023) [172]
	Positive aesthetic experience (1)	Sandberg et al. (2023) [181]
	Aesthetic experience (1)	Sutherland & Jelinek (2015) [22]
	Heightened aesthetic pleasure (1)	Parush & Koivunen (2014) [68]
	High aesthetic engagement (1)	Sutherland (2012) [183]
Satisfaction (5)	High satisfaction with course design (4)	Andenoro & Ward (2008) [166], Cranston & Kusanovich (2013) [167], Cranston & Kusanovich (2014) [168], Harz et al. (2023) [172]
	High satisfaction with assignment (1)	Hurdle & Greenhaw (2023) [173]
**Learning process (12)**		
Transformative learning (8)	Transformative learning (2)	Firing et al. (2022) [171], Woods et al. (2023) [45]
	Learner autonomy (1)	Rajendran & Andrew (2014) [51]
	Sustained growth (1)	Winther & Højlund Larsen (2022) [185]
	Effective learning tool (1)	Hurdle & Greenhaw (2023) [173]
	Vicarious learning (1)	Hurdle & Greenhaw (2023) [173]
	Memorable learning experiences (1)	Sutherland (2012) [183]
	Memorability (1)	Parush & Koivunen (2014) [68]
Comprehensive learning (4)	Enhanced memorization (1)	Rajendran & Andrew (2014) [51]
	Improved understanding (1)	Rajendran & Andrew (2014) [51]
	Contextual understanding (1)	Rajendran & Andrew (2014) [51]
	Practical learning experience (1)	Rajendran & Andrew (2014) [51]
**Sensory and experiential awareness (7)**		
Sensitivity (7)	Heightened sensory perception (1)	Katz-Buonincontro (2011) [50]
	Increased sensitivity (2)	Sandberg et al. (2023) [181], Winther (2018) [184]
	Awareness (1)	Harz et al. (2023) [172]
	Heightened sense of visual perception (1)	Katz-Buonincontro & Phillips (2011) [176]
	Increased observational skills (1)	Katz-Buonincontro et al. (2015) [177]
	Enhanced aesthetic awareness (1)	Woods et al. (2023) [45]

Table S3. *Cont.*

Emotional development and personal growth (33)		
Emotional awareness and transformation (11)	Enhanced emotional intelligence (1)	Feltham (2012) [170]
	Improved emotional intelligence (1)	Garavan et al. (2015) [69]
	Increased emotional competency (1)	Munro et al. (2015) [179]
	Enhanced emotional awareness (2)	Firing et al. (2022) [171], Winther & Højlund Larsen (2022) [185]
	Increased emotional awareness (1)	Sutherland (2012) [183]
	Heightened emotional awareness (1)	Munro et al. (2015) [179]
	Increased emotional engagement (1)	Leonard et al. (2013) [106]
	Emotional regulation (1)	Hirsch et al. (2023) [57]
	Emotional transformation (1)	Dennis (2014) [169]
	Emotional catharsis (1)	Katz-Buonincontro (2011) [50]
Self-awareness (12)	Enhanced self-awareness (2)	Dennis (2014) [169], Schyns et al. (2013) [35]
	Increased self-awareness (1)	Hirsch et al. (2023) [57]
	Improved self-awareness (2)	Romanowska et al. (2014) [79], Sutherland (2012) [183]
	Self-awareness (2)	Peña & Grant (2019) [180], Woods et al. (2023) [45]
	Somatic awareness (2)	Winther (2018) [184], Winther & Højlund Larsen (2022) [185]
	Improved self-contact (2)	Winther (2018) [184], Winther & Højlund Larsen (2022) [185]
	Increased awareness of preferences (1)	Munro et al. (2015) [179]
Empowered self-concept (10)	Increased confidence (1)	Feltham (2012) [170]
	Increased self-confidence (2)	Parush & Koivunen (2014) [68], Winther (2018) [184]
	Self-efficacy (1)	Peña & Grant (2019) [180]
	Better performance-based self-esteem (1)	Romanowska et al. (2011) [67]
	Increased personal agency (1)	Dennis (2014) [169]
	Increasing capacity for pro-active agency (1)	Woods et al. (2023) [45]
	Increased agency (1)	Kaimal et al. (2014) [174]
	Empowerment (2)	Kaimal et al. (2014) [174], Kaimal et al. (2016) [175]

Table S3. *Cont.*

**Reflective and reflexive practices (15)**		
Reflective and reflexive practices (15)	Reflective practice (2)	Dennis (2014) [169], Sutherland & Jelinek (2015) [22]
	Reflective thinking (1)	Katz-Buonincontro (2011) [50]
	Enhanced reflective practice (3)	Feltham (2012) [170], Kaimal et al. (2014) [174], Kaimal et al. (2016) [175]
	Improved reflective practice (1)	Harz et al. (2023) [172]
	Enhanced reflectivity (3)	Hirsch et al. (2023) [57], Katz-Buonincontro & Phillips (2011) [176], Kilic (2023) [40]
	Increased reflexivity (1)	Woods et al. (2023) [45]
Reflective and reflexive practices (15)	Improved reflexivity (1)	Winther & Højlund Larsen (2022) [185]
	Enhanced reflexivity (1)	Sutherland (2012) [183]
	Heightened self-reflection (1)	Schyns et al. (2013) [35]
	In-depth personal reflection (1)	Katz-Buonincontro et al. (2015) [177]
**Higher-order cognitive skills (14)**		
Reflective thinking (5)	Increased reflective thinking (2)	Andenoro & Ward (2008) [166], Cranston & Kusanovich (2013) [167]
	Improved reflective thinking (1)	Cranston & Kusanovich (2014) [168]
	Reflective thinking (1)	Katz-Buonincontro (2011) [50]
	Enhanced reflective learning (1)	Leonard et al. (2013) [106]
Critical thinking skills (3)	Improved critical thinking skills (1)	Andenoro & Ward (2008) [166]
	Improved critical thinking (1)	Singh & Widén (2020) [182]
	Improved problem-solving skills (1)	Katz-Buonincontro & Phillips (2011) [176]
Creativity (6)	Increased creativity (3)	Kaimal et al. (2014) [174], Katz-Buonincontro (2011) [50], Katz-Buonincontro & Phillips (2011) [176]
	Reconnection with creativity (1)	Katz-Buonincontro et al. (2015) [177]
	Enhanced creativity (1)	Kilic (2023) [40]
	Utilization of creative processes (1)	Hirsch et al. (2023) [57]

Table S3. *Cont.*

**Sense-making (12)**		
Sense-making (12)	Sense-making (2)	Peña & Grant (2019) [180], Sutherland & Jelinek (2015) [22]
	Disorienting dilemma (1)	Peña & Grant (2019) [180]
	Cultural exploration (1)	Dennis (2014) [169]
	Insight into leadership practice (1)	Katz-Buonincontro et al. (2015) [177]
	Recognition of essential leadership traits (1)	Singh & Widén (2020) [182]
	Holistic identity development (1)	Firing et al. (2022) [171]
	Deeper understanding of power and responsibility (1)	Sutherland & Jelinek (2015) [22]
	Leadership paradigm definition (1)	Katz-Buonincontro et al. (2015) [177]
	Broader perspective (1)	Harz et al. (2023) [172]
	Enhanced understanding (1)	Katz-Buonincontro et al. (2015) [177]
	Integration of body and mind (1)	Hirsch et al. (2023) [57]
**Adaptive resilience (19)**		
Negative capability (3)	Development of negative capability (1)	Hirsch et al. (2023) [57]
	Overcoming anxiety (1)	Firing et al. (2022) [171]
	Managing uncertainty (1)	Firing et al. (2022) [171]
Risk taking (5)	Increased risk taking (2)	Katz-Buonincontro & Phillips (2011) [176], Parush & Koivunen (2014) [68]
	Encouraged risk taking (1)	Katz-Buonincontro et al. (2015) [177]
	Increased willingness to take risks (1)	Leonard et al. (2013) [106]
	Relevance of risk taking (1)	Cranston & Kusanovich (2014) [168]
Mental health (11)	Better stress management (2)	Romanowska et al. (2013) [32], Romanowska et al. (2014) [79]
	Stress management (1)	Feltham (2012) [170]
	Reduction in stress indicators (1)	Romanowska et al. (2011) [67]
	Improved mental resilience (1)	Romanowska et al. (2013) [32]
	Improved mental health (1)	Romanowska et al. (2011) [67]
	Favorable biological outcomes (1)	Romanowska et al. (2011) [67]
	Enhanced coping strategies (1)	Romanowska et al. (2011) [67]
	Improved well-being (1)	Feltham (2012) [170]
	Wellbeing (1)	Harz et al. (2023) [172]
	Healing effect (1)	Kilic (2023) [40]

Table S3. *Cont.*

**Interpersonal and social competencies (35)**		
Empathy (8)	Enhanced empathy (4)	Andenoro & Ward (2008) [166], Cranston & Kusanovich (2013) [167], Harz et al. (2023) [172], Katz-Buonincontro (2011) [50]
	Increased empathy (4)	Cranston & Kusanovich (2014) [168], Feltham (2012) [170], Firing et al. (2022) [171], Kaimal et al. (2016) [175]
Ethical understanding (3)	Improved ethical understanding (1)	Cranston & Kusanovich (2013) [167]
	Enhanced ethical understanding (1)	Cranston & Kusanovich (2014) [168]
	Enhanced ethical humanist skills (1)	Medeiros et al. (2012) [178]
Communication skills (7)	Improved communication skills (1)	Kilic (2023) [40]
	Enhanced communication skills (1)	Winther & Højlund Larsen (2022) [185]
	Enhanced nonverbal communication (1)	Sandberg et al. (2023) [181]
	Improved communication (1)	Medeiros et al. (2012) [178]
	Adaptability in communication modes (1)	Munro et al. (2015) [179]
	Enhanced communication effectiveness (1)	Munro et al. (2015) [179]
	Increased feedback orientation (1)	Garavan et al. (2015) [69]
Interpersonal skills (9)	Improved interpersonal skills (1)	Feltham (2012) [170]
	Improved interpersonal efficacy (1)	Dennis (2014) [169]
	Development of relational skills (1)	Winther & Højlund Larsen (2022) [185]
	Enhanced humanistic qualities (1)	Sutherland & Jelinek (2015) [22]
	Heightened awareness of relational dynamics (1)	Sutherland & Jelinek (2015) [22]
	Enhanced social awareness (1)	Firing et al. (2022) [171]
	Interpersonal awareness (1)	Woods et al. (2023) [45]
	Increased social sensitivity (1)	Kilic (2023) [40]
	Enhanced perceptual alignment (1)	Romanowska et al. (2014) [79]
Collaboration (8)	Improved collaboration (1)	Leonard et al. (2013) [106]
	Improved teamwork (2)	Leonard et al. (2013) [106], Medeiros et al. (2012) [178]
	Enhanced collaborative decision-making (1)	Cranston & Kusanovich (2014) [168]
	Community building (1)	Firing et al. (2022) [171]
	Sense of community (2)	Katz-Buonincontro (2011) [50], Kilic (2023) [40]
	Improved collaborative leadership capabilities (1)	Woods et al. (2023) [45]

Table S3. *Cont.*

**Comprehensive leadership development (12)**		
Leadership perspective (5)	Broader leadership perspective (1)	Andenoro & Ward (2008) [166]
	Broadened perspectives on leadership (2)	Kaimal et al. (2014) [174], Kaimal et al. (2016) [175]
	Changed leadership perspectives (1)	Singh & Widén (2020) [182]
	Widening perspectives on leadership (1)	Woods et al. (2023) [45]
Leadership enhancement (4)	Improved leadership (1)	Medeiros et al. (2012) [178]
	Enhanced leader identity (1)	Garavan et al. (2015) [69]
	Improved leader performance (1)	Romanowska et al. (2014) [79]
	Development of practical skills (1)	Cranston & Kusanovich (2014) [168]
Embodied leadership (3)	Increased embodied leadership competence (1)	Winther & Højlund Larsen (2022) [185]
	Embodied leadership (1)	Winther (2018) [184]
	Improved physical presence (1)	Sandberg et al. (2023) [181]
**Transfer success (14)**		
Real-world application (9)	Real-world application of theories (1)	Andenoro & Ward (2008) [166]
	Real-world application (1)	Cranston & Kusanovich (2013) [167]
	Practical application (1)	Singh & Widén (2020) [182]
	Long-term impact on professional practice (1)	Sutherland & Jelinek (2015) [22]
	Reconsidering future leadership practice (1)	Sutherland (2012) [183]
	Successful learning transfer (1)	Sandberg et al. (2023) [181]
Real-world application (9)	Transfer to professional context (1)	Kaimal et al. (2016) [175]
	Learning transfer to real world (1)	Leonard et al. (2013) [106]
	Increased innovation (1)	Kaimal et al. (2014) [174]
Behavioral change (5)	Behavioral change (1)	Feltham (2012) [170]
	Enhanced pro-social behavior (1)	Romanowska et al. (2013) [32]
	Reduced passive leadership (1)	Romanowska et al. (2013) [32]
	Reduction in passive leadership (1)	Romanowska et al. (2014) [79]
	Positive impact on subordinates (1)	Romanowska et al. (2014) [79]

Note. Numbers in round brackets indicate the number of reports contributing to a theme.
